# Unraveling dual feeding associated molecular complexity of salivary glands in the mosquito *Anopheles culicifacies*

**DOI:** 10.1242/bio.012294

**Published:** 2015-07-10

**Authors:** Punita Sharma, Swati Sharma, Ashwani Kumar Mishra, Tina Thomas, Tanwee Das De, Suman Lata Rohilla, Namita Singh, Kailash C. Pandey, Neena Valecha, Rajnikant Dixit

**Affiliations:** 1 Host-Parasite Interaction Biology Group, National Institute of Malaria Research, Sector-8, Dwarka, Delhi 110077, India; 2 Nano and Biotechnology Department, Guru Jambheshwar University, Hisar, Haryana 125001, India; 3 NxGenBio Lifesciences, C-451, Yojna Vihar, Delhi 110092, India

**Keywords:** Malaria, Mosquito, Salivary gland, Sugar and blood feeding, Gene expression

## Abstract

Mosquito salivary glands are well known to facilitate meal acquisition, however the fundamental question on how adult female salivary gland manages molecular responses during sugar versus blood meal uptake remains unanswered. To investigate these responses, we analyzed a total of 58.5 million raw reads generated from two independent RNAseq libraries of the salivary glands collected from 3–4 day-old sugar and blood fed *Anopheles culicifacies* mosquitoes*.* Comprehensive functional annotation analysis of 10,931 contigs unraveled that salivary glands may encode diverse nature of proteins in response to distinct physiological feeding status. Digital gene expression analysis and PCR validation indicated that first blood meal significantly alters the molecular architecture of the salivary glands. Comparative microscopic analysis also revealed that first blood meal uptake not only causes an alteration of at least 12–22% of morphological features of the salivary glands but also results in cellular changes e.g. apoptosis, confirming together that adult female salivary glands are specialized organs to manage meal specific responses. Unraveling the underlying mechanism of mosquito salivary gene expression, controlling dual feeding associated responses may provide a new opportunity to control vector borne diseases.

## INTRODUCTION

Sugar feeding by adult mosquitoes is not only essential for regular metabolic energy production, but also required to maintain a wealth of behavioral, structural, and physiological demands to survive in diverse habitats (Foster, 1995). However blood feeding by adult female mosquitoes is essential to meet the extra nutrient requirement for egg production and life cycle maintenance. Thus blood and sugar feeding are mutually exclusive and antagonistic behavioral-cum-physiological properties of conflicting demands (Foster, 1995), making it hard to understand the biological consequences of the mosquito tissues involved in feeding and digestion.

Although, evolutionary adaptive responses to blood feeding in mosquitoes remains largely unknown, it is believed that this may have arisen independently 145–165 million years ago (Balashov, 1984; Ribeiro et al., 1995). Furthermore, adaptation to blood meal acquisition from vertebrate hosts by adult females might have favored the evolution of specialized feeding organs including the proboscis (Maekawa et al., 2011) and enlarged salivary glands (James, 2003). In fact during blood meal uptake adult female salivary glands also play a key role to facilitate the entry and exit of pathogens (Das et al., 2010; Dhar and Kumar, 2003; Dixit et al., 2009; James, 2003; Ribeiro et al., 2010; Rodriguez and Hernandez-Hernandez, 2004). Morphologically, adult female salivary glands are a well specialized pair of tri-lobed single layered epithelial tissues able to synthesize and release a battery of diverse molecules including anti-haemostatic factors e.g. anticoagulants, vasodilators, sialokinins etc. into the skin of the host, for successful blood meal uptake (Dhar and Kumar, 2003; James, 2003; Nieves et al., 2012). For the last decade, multiple transcriptomic and proteomic studies have been valuable to identify such mosquito salivary factors (Ribeiro et al., 2010) but several fundamental questions in relation to the evolution of the dual feeding behavior ‘in general’ and functional role of salivary glands before and after blood meal uptake ‘in specific’, remains unanswered. Finding such key molecular factors could be crucial to manipulate mosquito behavior by interfering with mosquito feeding and hence the parasite transmission (Cator et al., 2013; Jacobs-Lorena, 2003).

In nature, any successful feeding event by the mosquito needs to be dealt with multiple and sequential behavioral coordinates e.g. searching, locating, landing and probing the suitable host whether plant or vertebrate (Takken and Verhulst, 2013). Thus how adult female mosquito salivary glands manage meal specific responses remains largely unknown. Recent micro-array based salivary transcriptome analysis, predicts at least ninety three putative transcripts for which expression altered significantly in response to a blood meal in *Anopheles gambiae* (Das et al., 2010). Although valuable, but microarray strategy solely relies on genome based predicted transcripts and could easily miss the information for the rare sequences that remains un-annotated or expressed below threshold level (Zhao et al., 2014). In recent years, next-generation sequencing not only opened the door for functional genomics analysis, but also emerged as an important tool to understand the evolutionary relationship of the molecular codes identified from non-model organisms (Bao et al., 2012; Gibbons et al., 2009; Hittinger et al., 2010; Su et al., 2012; Wang et al., 2010). Furthermore, deep sequencing RNAseq technologies may also facilitate in depth annotation of the draft genome sequence available for multiple anopheline mosquito species (www.vectorbase.org) (Gomez-Diaz et al., 2014; Padrón et al., 2014).

Currently, we are trying to understand molecular relationships of the salivary glands controlling dual feeding behavior and *Plasmodium* transmission (Dixit et al., 2011, 2009). In the present investigation we focused on the molecular composition and possible functional relationships of salivary factors changing under two physiologically distinct feeding status i.e. naïve sugar fed to first blood meal in the mosquito *A. culicifacies*, an important rural malarial vector which transmits more than 65% malaria in India (Dev and Sharma, 2013; Goswami et al., 2006). Our RNAseq based comparative salivary transcriptomic analysis provides evidence that adult female mosquitoes have the unique ability to regulate meal specific molecular and cellular responses.

## RESULTS AND DISCUSSION

A successful feeding event by mosquito depends on several behavioral coordinates/stimuli viz. suitable host searching, locating, landing, proper feeding site selection, proboscis punching and meal specific salivary actions/responses. In contrast to sugar feeding by both sexes, the adult female mosquito also take vertebrate blood meal for reproductive success. Thus how adult female mosquito manages this complex behavioral process to finalize meal specific choices/decisions, and directs salivary glands for completion of feeding event, remains largely unknown. To closely mimic the natural feeding behavior of a young adult female mosquito seeking its first blood meal, we planned to know whether mosquito blood feeding preference to a particular vertebrate host (rabbit in the present study) is a ‘random’ or ‘specific’ process, especially when both meal sources are accessible at the same time. Considering different physiological and feeding status of the mosquitoes, we performed multiple blood feeding preference experiments (supplementary material Fig. S1). We also tested blood meal preference when both meals were offered together to unstarved (on regular sugar meal) mosquitoes, but no significant differences could be observed in the blood meal preference (supplementary material Fig. S1). Taken together, we concluded that any dual feeding associated food choice is a ‘flexible/random’ behavioral process, probably guided by multiple internal factors associated with age, mating, feeding and innate physiological status of individual mosquito (Takken and Verhulst, 2013). The knowledge on the molecular relationships of salivary glands under dual feeding status is very limited. Unraveling such relationships is not only challenging but also crucial to understand how salivary glands facilitate conflicting demands, if both meal sources are available. We hypothesize that salivary gland must have a unique ability to manage meal specific molecular responses to facilitate successful meal acquisition.

To test this hypothesis, first we chose to carry out dual feeding experiments with the 3−4 day-old naïve adult female mosquitoes reared on the synthetic sugar and rabbit (blood source). Assuming similar feeding status of the naïve adult female mosquitoes in nature, we collected salivary tissues from two groups separated from the same cohort of un-starved mosquitoes (i) that remains on sugar meal (ii) that is successfully able to take the first blood meal from rabbits when offered (supplementary material Fig. S1). To trace the possible molecular factors changing under dual feeding status i.e. prior and post first blood meal, we adopted an Illumina based deep sequencing approach as a proof of concept for gene discovery. We sequenced two RNAseq libraries prepared independently from a minimum of thirty five pairs of salivary glands pooled from 3–4 day-old ‘naïve sugar fed’ or immediate ‘blood’ fed (<1 h) adult female mosquitoes collected from the same cohorts of the mosquito cage.

The above protocol in fact generated a total of ∼58.5 million raw reads, which were quality filtered and *de novo* assembled, yielding a set of 10,931 [5241 for sugar fed (SF) and 5690 for blood fed (BF) library] contigs. Initially, the quality of the *de novo* assembly was carefully examined by multiple homology search analysis of the whole transcriptome dataset against draft genome/transcript databases for the mosquito *A. culicifacies*, available at www.vectorbase.org. As expected 95% transcripts yielded significant match (10^−5^ e-value) to the draft genome of the mosquito *A. culicifacies*, at nucleotide level, proving no annotation bias. Later, we selected few full length cDNA transcripts (>1000 bp) and compared them with previously well annotated genes identified from other mosquitoes (supplementary material Fig. S2, S3, Table S1). Subsequent validation of the selected transcripts by RT-PCR based expression analysis not only confirmed the quality of the assembly, but also allowed us to find out those rare transcripts which remained previously un-noticed, as mentioned in the text below. Detailed stats of the salivary transcriptome assembly kinetics have been summarized in Table 1.

**Table 1.**
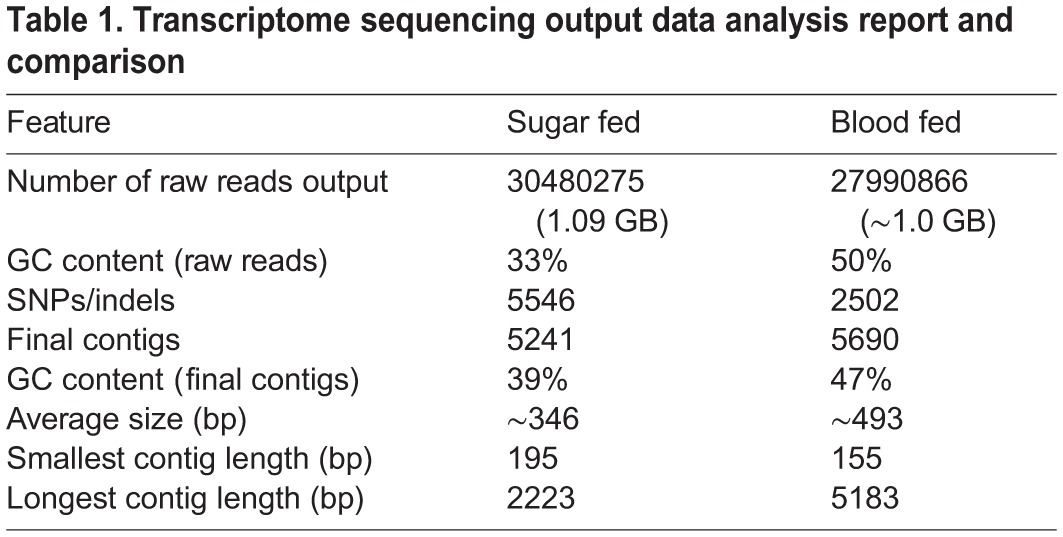
**Transcriptome sequencing output data analysis report and comparison**

### Salivary gland encodes diverse proteins in response to dual feeding

**Fig. 1.**
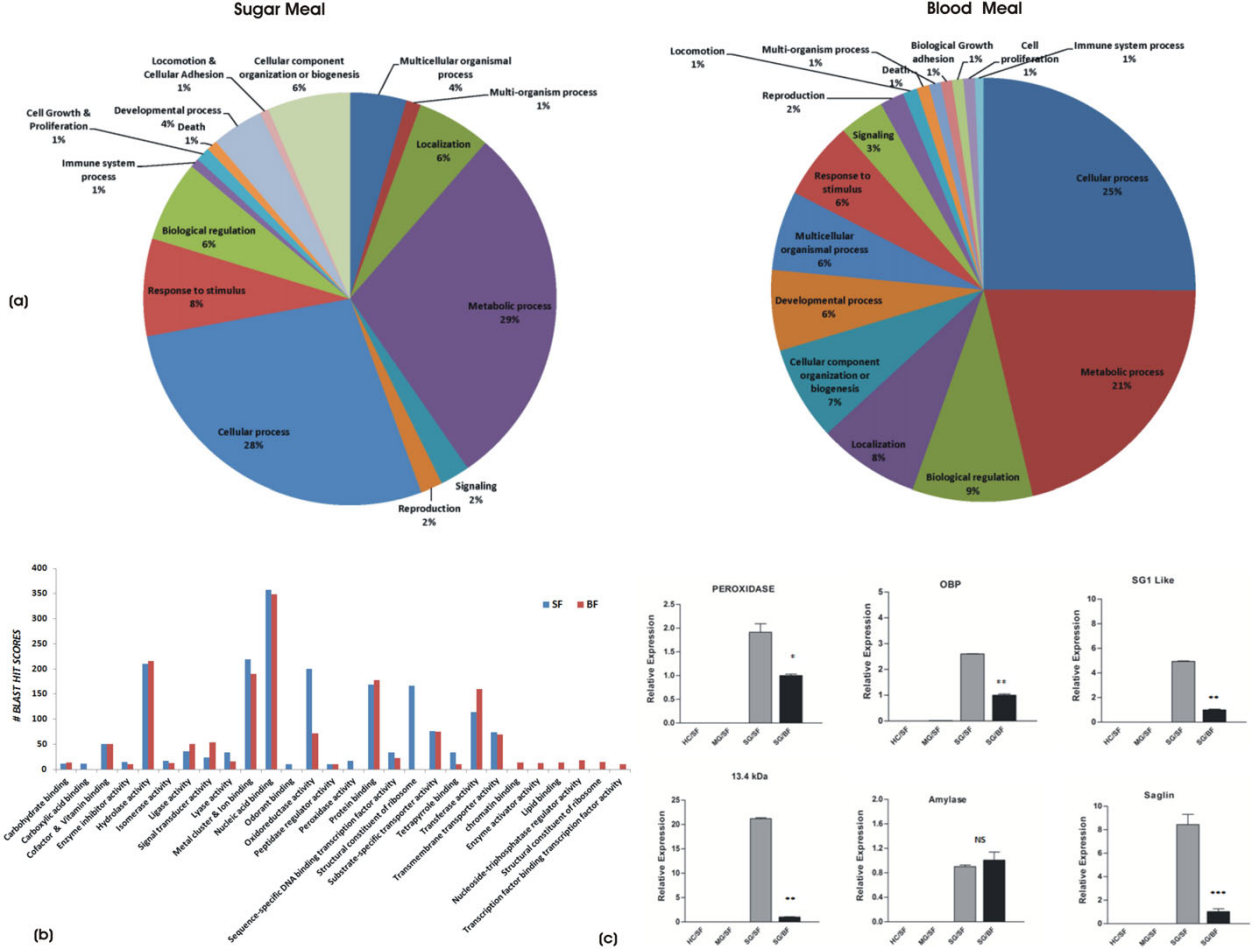
**Salivary glands encode diverse** **proteins.** (A) Functional classification and distribution of mosquito salivary encoded proteins identified from the individual transcriptome databases. (B) Comparative analysis of GO term classification (level 3) of salivary proteins engaged in diverse/unique functions. (C) experimental verification of salivary genes: tissue specific and differential regulation of randomly selected transcripts in response to blood feeding (*P≤0.05; **P≤0.001); HC, Hemocyte; MG, midgut; SG, salivary gland; SF, sugar fed; BF, blood fed. Error bar represents standard deviation from three biological replicates.

To understand the biological meaning of these putative salivary transcripts repertoires, we first performed a comprehensive functional prediction analysis for both datasets, by using online BLAST2GO software. In contrast to 59% best match of the blood fed salivary transcript, only 21% of sugar fed salivary transcripts showed significant match to the available NR databases (supplementary material Table S4). This underscores our limited knowledge on the molecular nature of the salivary genes that assist in sugar meal acquisition/digestion on routine basis in naïve mosquitoes (Calvo et al., 2008; Dixit et al., 2011; Grossman and James, 1993). To clarify whether these un-annotated/unmatched sequences of sugar fed library origin, really express in salivary glands or appeared as an artifact of sequencing database assembly, we randomly selected at-least 10 hypothetical/unmatched sequences and performed RT-PCR analysis. Out of 10 at least 8 transcripts showed amplification, confirming that observed large scale un-annotated sequences may code some functional proteins (supplementary material Fig. S3). Unexpectedly, we also identified a cluster of salivary transcripts encoding plant like proteins, whose nature and origin is yet to be established, and therefore, we excluded these sequences from the current study. We believe future investigations involving large scale NGS based transcriptomic as well as full annotation of genomes may clarify this complexity. Here, we compared all the dataset against multiple protein databases as described earlier (Dixit et al., 2009). As expected, this analysis showed very distinct functional feeding dynamics for each library dataset (Table 2, Fig. 1 and supplementary material Fig. S4), indicating that salivary glands may encode distinct family proteins of diverse nature under dual feeding status.

**Table 2.**
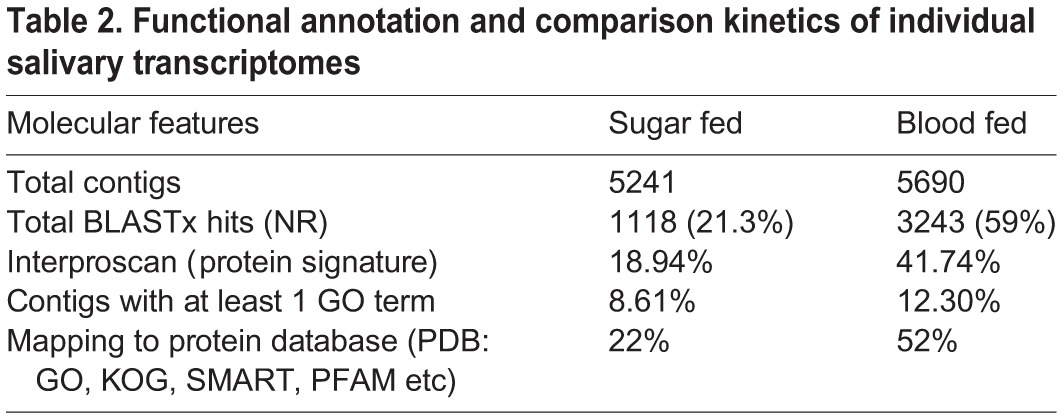
**Functional annotation and comparison kinetics of individual salivary transcriptomes**

To predict functional relationship, we manually short listed the BLAST hit scores of the GO terms (classified at level 3) and compared common/unique salivary transcripts associated with diverse molecular and cellular functions of salivary glands under distinct physiological feeding status (Fig. 1B). In total 28 GO terms could be assigned to the combined transcript dataset that showed the best match to the PDB and NR databases. A comparative analysis further revealed that at least 21 terms are common to both sugar fed and blood fed salivary glands, probably dealing with common housekeeping structural, molecular and biochemical functions e.g. energy metabolism, proteins synthesis and cellular machinery components and their maintenance, signal transduction, hydrolases, lyases etc. Three GO terms remain uniquely associated with sugar fed (odorant binding, carboxylic acid binding and peroxidase binding proteins), and six with blood fed (chromatin binding, structural constituents of ribosome, lipid binding, enzyme activator activity, nucleoside-triphosphatase regulator activities and transcription factor binding activity probably regulating meal specific salivary functions) (Fig. 1B).

For successful blood meal uptake, the undergoing salivary changes may cause direct recruitment of a large pool of secretary proteins to be delivered inside the body of the vertebrate host, resulting in the depletion of salivary protein content (Golenda et al., 1995). Although, tracking the ongoing molecular events under dual feeding status remains a challenge, but here we classified the putative salivary glands proteins likely engaged in the facilitation of meal acquisition and digestion. To do this we extensively performed a homology search analysis against reference databases, available for other salivary transcriptomes of the mosquito *Anopheles gambiae*, *Anopheles stephensi*, *Aedes aegypti* and *Culex quinquifaciatus* (Arca et al., 2005; Calvo et al., 2004, 2010; Das et al., 2010; Dixit et al., 2011, 2009; Ribeiro et al., 2004), and manually shortlisted/catalogued the molecular repertoire of unique salivary family protein expressing in response to dual feeding (Table 3).

**Table 3.**
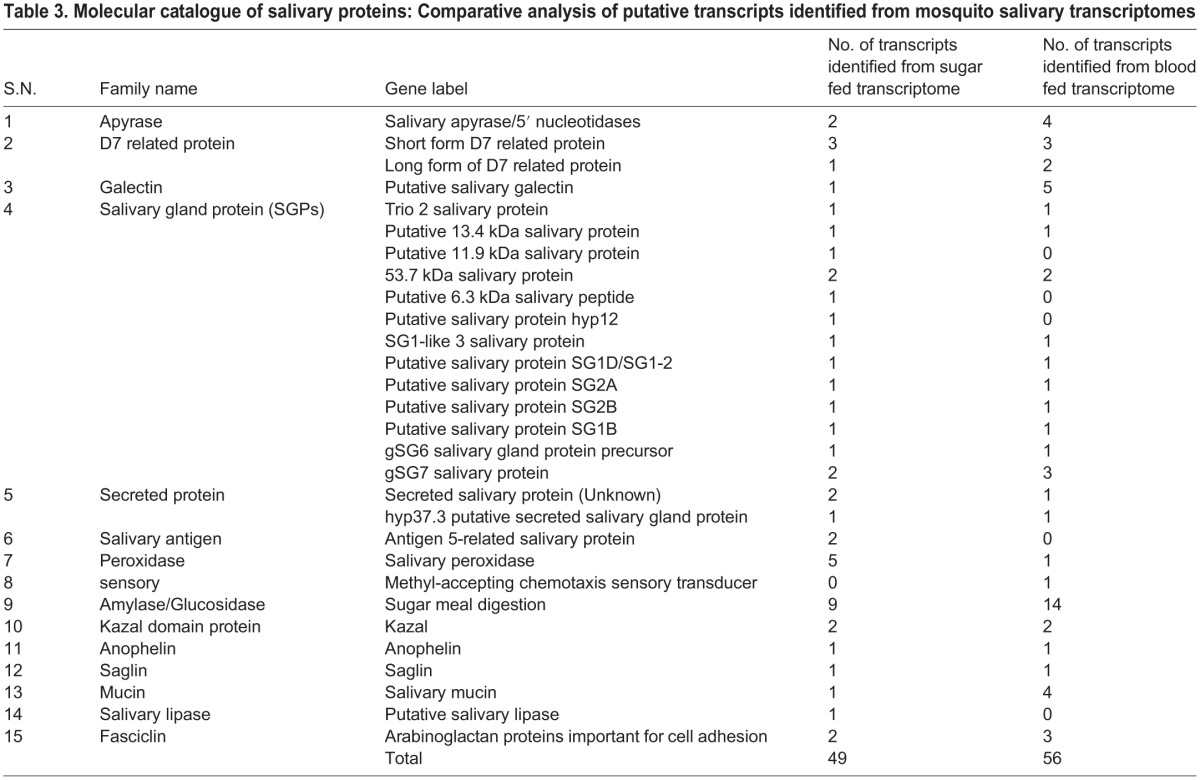
**Molecular catalogue of salivary proteins: Comparative analysis of putative transcripts identified from mosquito salivary transcriptomes**

A total of 105 salivary transcripts encoding enzymes and secretary proteins could be distributed into 15 family proteins. Interestingly, in this analysis we not only observed overall changes in the common salivary transcript numbers, but also identified several meal specific unique salivary transcripts. Notably, these included subclasses of salivary gland proteins (SGPs); peroxidases, salivary antigens; mucin etc. (Table 3). Our tissue specific real-time PCR based analysis experimentally validated the molecular nature of salivary origin and also confirmed their differential response under distinct physiological status of feeding (Fig. 1C).

### Analysis of salivary immunome: feeding associated complexity of local immune response

**Fig. 2.**
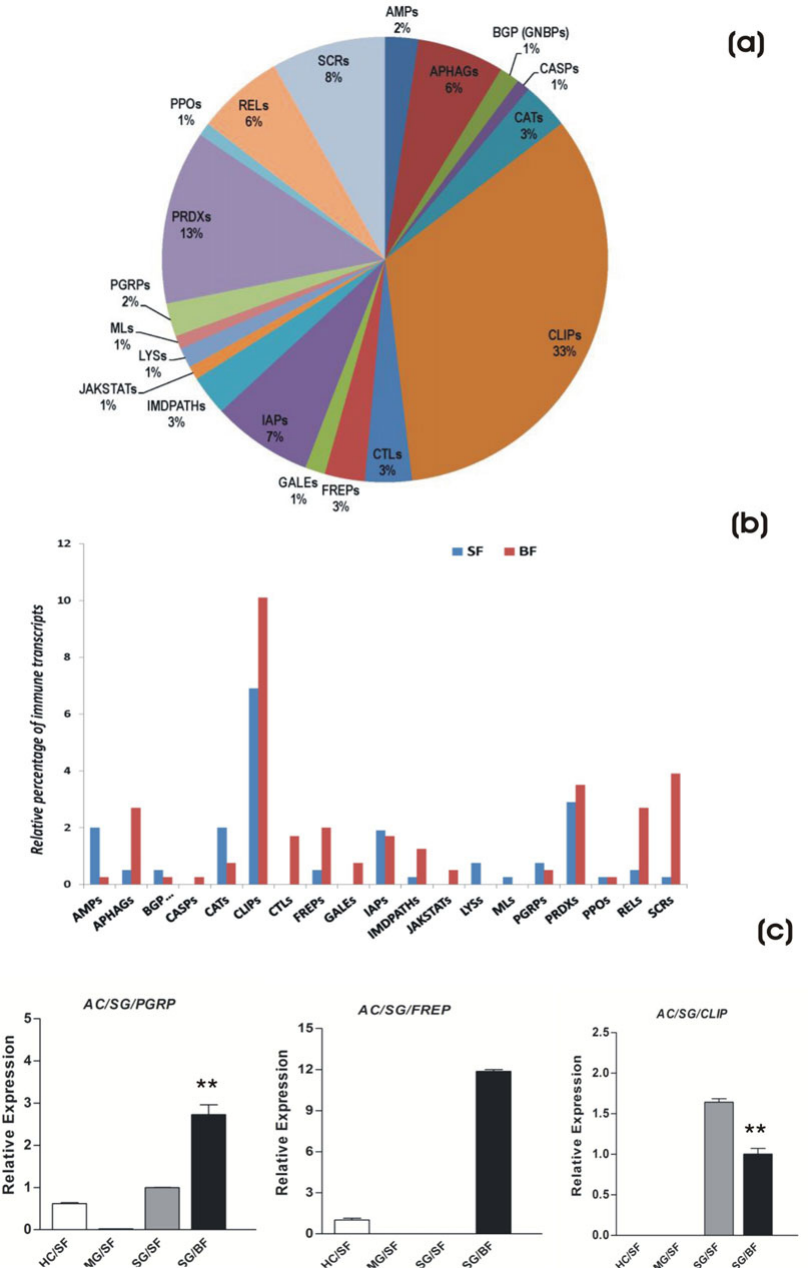
**Molecular characterization of mosquito salivary immunome.** (A) Molecular catalog of putative salivary immune transcripts predicted from the salivary gland transcriptome. (B) Change in the relative percentage of immune transcripts in response to differential feeding. (C) Tissue specific and differential regulation of the selected immune transcripts in response to blood feeding (*P≤0.05; **P≤0.001); HC, Hemocyte; MG, midgut; SG, salivary gland; SF, sugar fed; BF, blood fed. Error bar represents standard deviation from three biological replicates.

From early development, mosquitoes are regularly exposed to diverse microbes during feeding; a healthy status is maintained through active ‘local’ and ‘systemic’ immune responses (Ponton et al., 2013). Although several putative immune transcripts have been identified from the salivary transcriptomes of different mosquito species (Dixit et al., 2009, 2008; Ribeiro et al., 2010), we still have very limited knowledge of the immune transcripts differentially expressed in response to dual feeding. In the current study, we screened and compared whole salivary transcriptome against insect ImmunoDB database available at http://cegg.unige.ch/insecta/immunodb/, allowing us to identify a total of 204 salivary transcripts that could be classified into 19 immune family proteins (Fig. 2A).

Comparatively, we observed increased number of transcripts in the blood fed salivary glands (130 transcripts) as compared to sugar fed mosquitoes (74 transcripts). A difference was observed in the number for the members of Autophagy (APHAGs), clip domain serine proteases (CLIPs), peroxidase (PRDX), fibrinogen related proteins (FREPs), relish like proteins (RELs), inhibitors of apoptosis (IAPs), peptidoglycan recognition proteins (PGRPs), scavenger receptor (SCRs) etc. family proteins in response to blood feeding, while a few other members of the immune transcripts e.g. c-type lectins (CTLs), galactoside-binding lectins (GALEs), lysozymes (LYS) etc., showed meal specific restricted expression, revealing a unique change in the salivary immunome (Fig. 2B).

Our DGE data analysis, as described in ‘Salivary gene expression switching manages meal specific responses’, revealed at least 40 immune transcript's expression, significantly altered in response to differential feeding (supplementary material Table S2), notably up regulation of CLIP members, while down regulation of AMPs in response to blood feeding. To verify the observation we examined the expression of few selected immune transcripts by real-time PCR analysis (Fig. 2C), together, suggesting that immune components may also participate in some physiological responses in meal uptake by the salivary glands (Gulley et al., 2013).

The constitutive synthesis and secretion of salivary immune proteins e.g. cecropins, defensin, GNBPs may facilitate proper meal ingestion and digestion, maintaining tissue sterility (Dixit et al., 2008; Godreuil et al., 2014). Recently, gut associated microbial flora has been shown to influence diverse cellular functions including food digestion, metabolism and vector competence (Minard et al., 2013), however, the role of salivary flora, especially in relation to sugar feeding, digestion and immunity remains unknown. Our recent finding of more diverse microbial community to salivary glands than gut (Sharma et al., 2014), suggests that regular interaction of salivary flora may also influence meal acquisition, digestion and local immune response against microbial pathogens including parasites and viruses (Gimonneau et al., 2014; Minard et al., 2013).

### Deep sequencing revealed novel salivary transcripts

Over a decade, a series of salivary transcriptomes have been characterized from different insects, including mosquito vectors allowing comprehensive cataloging of common and species specific salivary proteins (Ribeiro et al., 2010). To uncover underlying molecular and evolutionary relationship of salivary products, there is always an interest to discover new molecules from every transcriptome. In the current study we not only identified previously well described salivary transcripts (Table 3), but also found new transcripts, previously uncharacterized from anopheline mosquito species. Notably we characterized a new putative transcript encoding a kunitz domain, 23.4 kDa, Cystatin and other hypothetical proteins, from the mosquito *A. culicifacies* salivary glands.

#### Analysis of novel 23.4 kDa mosquito specific salivary protein

**Fig. 3.**
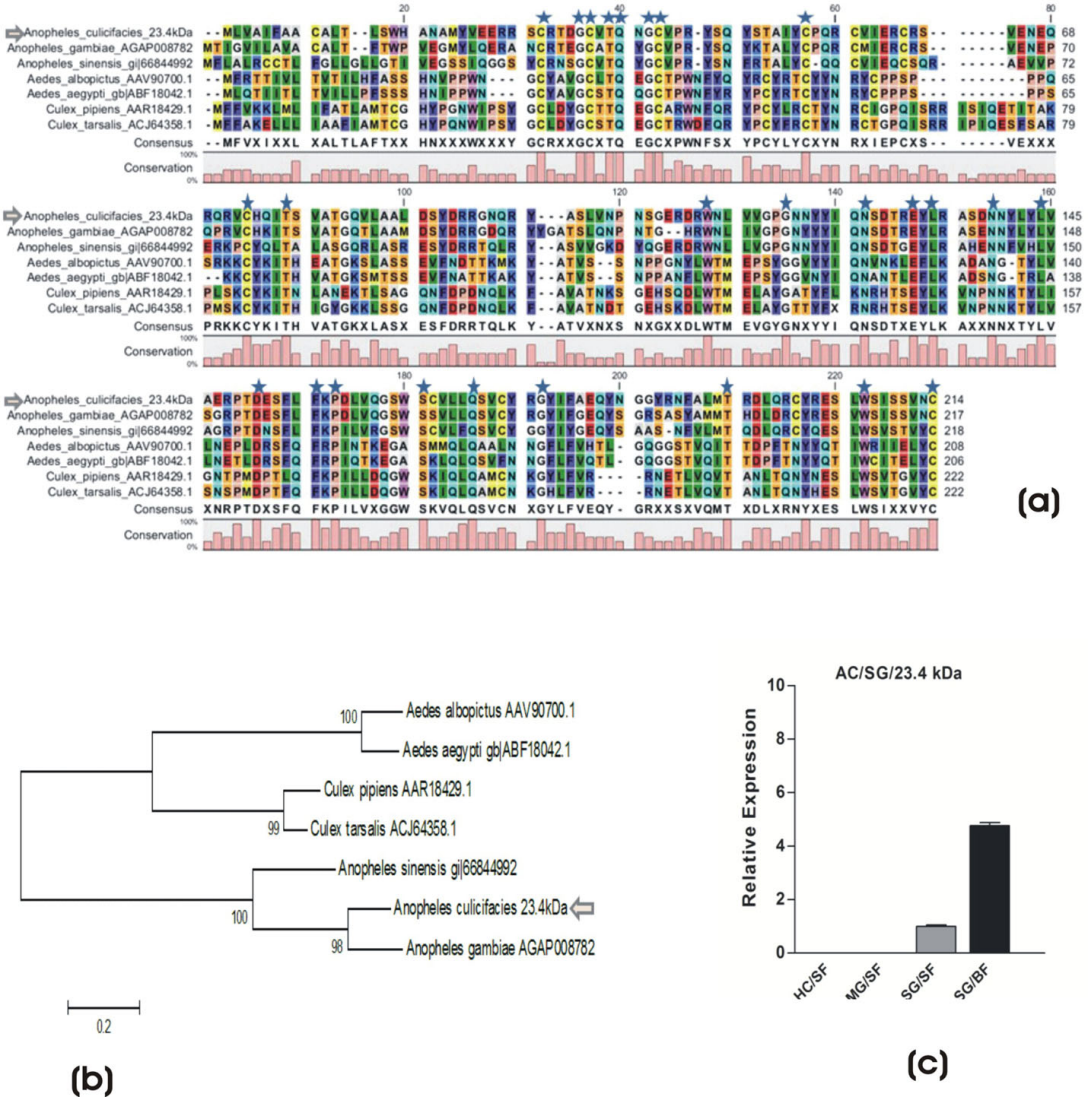
**Molecular analysis of novel 23.4 kDa mosquito specific salivary protein**, (A) Multiple sequence and (B) phylogenetic analysis, revealing identical features and conserved relationship to the previously reported non-anopheline mosquito-specific 23.4 kDa salivary proteins and genome predicted mosquito proteins. Conserved residues have been marked with a blue star. Tissue specific and differential regulation of the selected immune transcripts in response to blood feeding; HC, hemocyte; MG, midgut; SG, salivary gland; SF, sugar fed; BF, blood fed. Error bar represents standard deviation from three biological replicates.

For blood meal uptake, salivary gland releases large number of salivary specific anti-haemostatic factors, that are conserved in most of the blood feeding insects/mosquitoes (Ribeiro and Francischetti, 2003; Ribeiro et al., 2010). However, there are few salivary proteins e.g. 23.4 kDa family protein known to be restricted to *Aedes* and *Culex* mosquitoes only. For the first time, we identified a 988 bp long full length salivary transcript, encoding a 214 amino acid cysteine-rich protein from the mosquito *A. culicifacies,* which showed a weak similarity to 23.4 kDa salivary secretary protein of *Aedes* (27–30% identity; E-value=1e^−11^ and *Culex* (32–33% identity; E-value=4e^−25^) mosquitoes (supplementary material Fig. S5) and higher similarity to genome predicted sequences from *A. gambiae* (75% identity; E-value=4e^−114^) and *A. sinensis* (66% identity; E-value=4e^−79^) mosquitoes (supplementary material Fig. S5). Multiple iterations of Psi-blasT with NR databases were able to assemble only 23.4 kDa salivary or genome predicted hypothetical proteins of blood feeding mosquito species. The multiple sequence alignment (Fig. 3A) and phylogenetic analysis (Fig. 3B) suggest that either this unique family protein is evolved *de novo* and co-opted as salivary protein within the mosquito species or they may have common ancestor, which remains unknown. Our real-time PCR based expression analysis revealed that this protein is exclusively secreted in the salivary glands and significantly (*P*≤0.0001) up-regulated in response to blood feeding, suggesting their role in blood meal uptake from vertebrae host (Fig. 3C).

#### Analysis of novel KUNITZ domain related salivary protease inhibitor

**Fig. 4.**
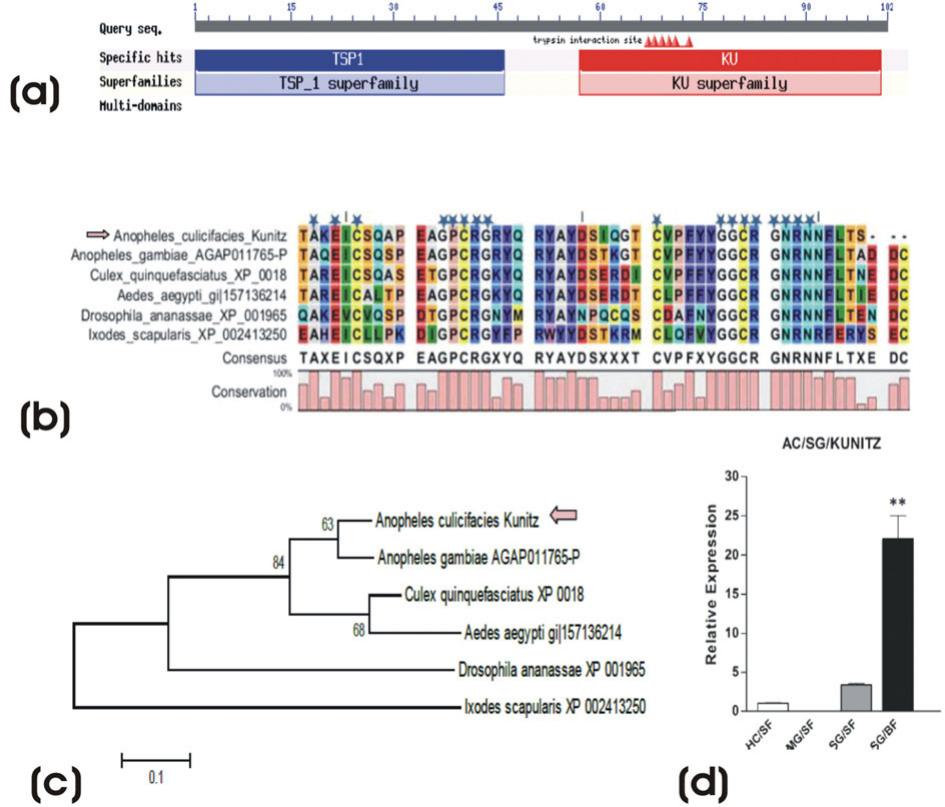
**Molecular analysis of novel salivary transcript encoding kunitz like protease inhibitor.** (A) Web based identification of a salivary transcript encoding bi-functional thrombospondin (TSP) and Kunitz/BPTI domains, previously remains un-described from any mosquito species. The multiple sequence alignment (B) and phylogenetic (C) analysis of the selected Kunitz domain with identical features of a typical Kunitz/BPTI domain carrying cysteine (yellow color) pattern of CX(8)CX(15)CX(7)CX(12)CX(3)C. The predicted Kunitz sequence of ticks was considered as out group for the construction of N/J tree at 1000 boot strap values. (D) Up-regulation of salivary Kunitz (**P≤0.005) in response to blood feeding. Error bar represents standard deviation from three biological replicates.

Kunitz/BPTI proteins (protease inhibitor) have been found to be abundantly expressed in tick salivary glands, likely play important role in blood feeding for longer duration (Dai et al., 2012). Although, Kunitz multi-domain proteins have been predicted from the genome of the mosquitoes, however, so far salivary specific Kunitz remain un-described from the salivary glands of the mosquitoes. We identified a putative 307 bp partial cDNA sequence encoding a protein with at least two (bi-functional) thrombospondin (TSP) and Kunitz (BPTI) like domains from the salivary gland transcriptome of the mosquito *A. culicifacies* (Fig. 4A). Sequence alignment and phylogenetic analysis of the selected Kunitz domain protein revealed identical features of a typical Kunitz/BPTI domain carrying cysteine (yellow color) pattern of CX(8)CX(15)CX(7)CX(12)CX(3)C and conserved phylogenetic relationship with the predicted mosquito Kunitz, likely originated from tick (Fig. 4B,C). Although, in other mosquitoes, the role of predicted Kunitz members is not known, however, salivary specific expression and significant up-regulation (*P*≤0.005) in response to blood feeding (Fig. 4D), suggest that the Ac-Kunitz may play important role in blood feeding of the mosquito.

#### Molecular analysis of salivary cystatin protein

**Fig. 5.**
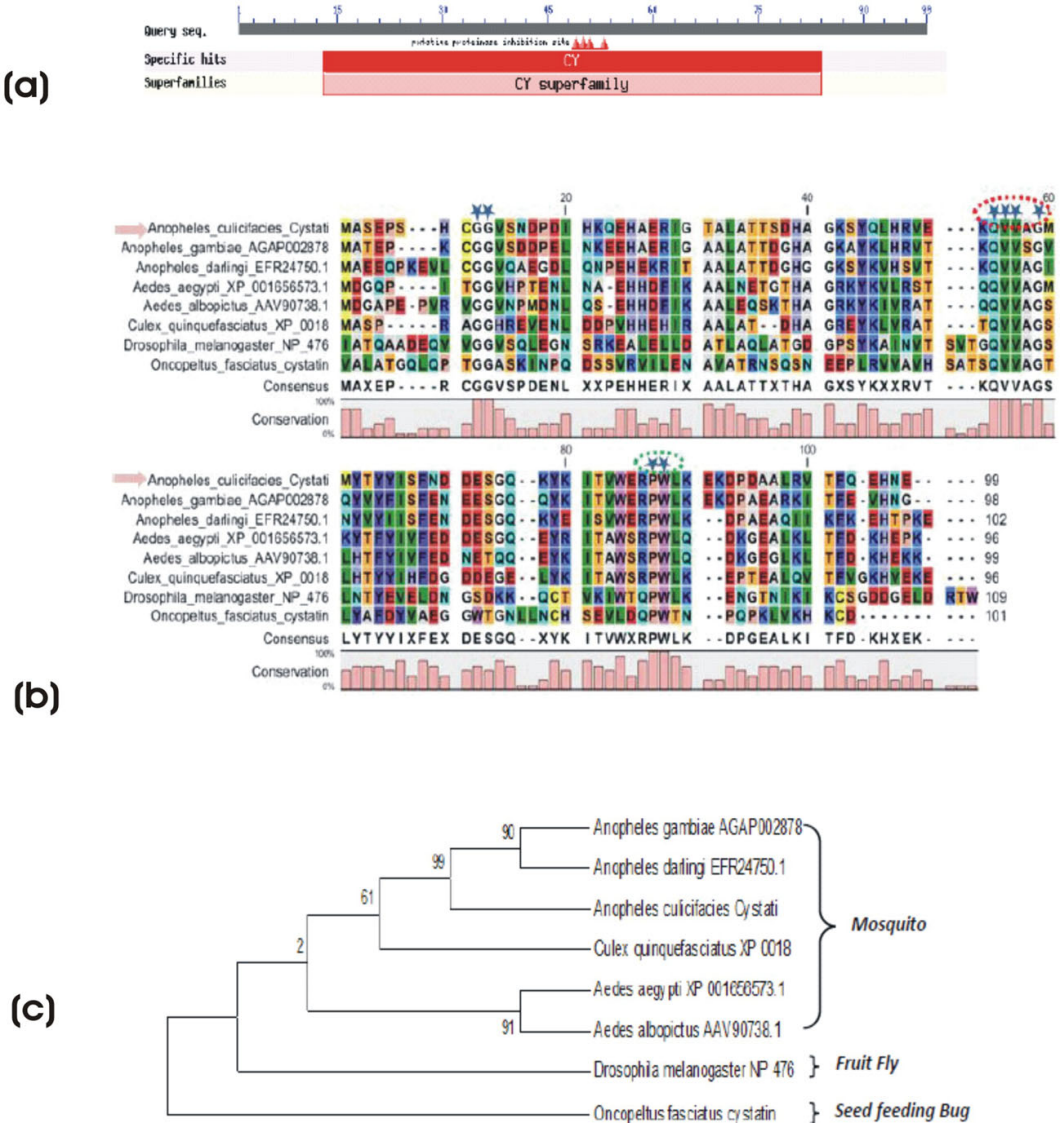
**Molecular analysis of salivary cystatin protein.** (A) Web prediction of salivary transcripts encoding cystatin like proteins of the insect. (B) Multiple sequence alignment of insect cystatins containing conserved motif QXVXG (red circle) and the dipeptide PW (green circle) near the carboxy terminus. (C) Phylogenetic relationship of putative salivary cystatin.

Cystatin proteins have been implicated for their specific inhibition of cysteine proteases (Zavasnik-Bergant, 2008). Although, an expanded family members of cystatin have been identified from salivary glands of the seed feeding bug (*Oncopeltus faciatus*) (Francischetti et al., 2007), they however, remains uncharacterized from any mosquito species. Interestingly, we identified a unique salivary transcript (464 bp long) from sugar fed library (Fig. 5A), which showed low identity (35–59%) to the other predicted cystatin like proteins of the insect and mosquitoes. All aligned insect cystatins contain signal peptides, indicative of secretion, and also showed the signature sequences of the conserved motif QXVXG (red circle) and the di-peptide PW (green circle) near the carboxy terminus (Fig. 5B). Phylogenetic analysis revealed conserved relationship of Ac-salivary cystatin to the genome predicted mosquito-cystatins, and divergence with fly and seed feeding insect (Fig. 5C). Although, a functional relationship of the putative salivary cystatin have yet to be established, our current analysis predict that insect-cystatin might be originated from plant feeding insects, to regulate cysteine proteases activity associated with sugar feeding.

#### Analysis of other salivary proteins involved in dual feeding

**Fig. 6.**
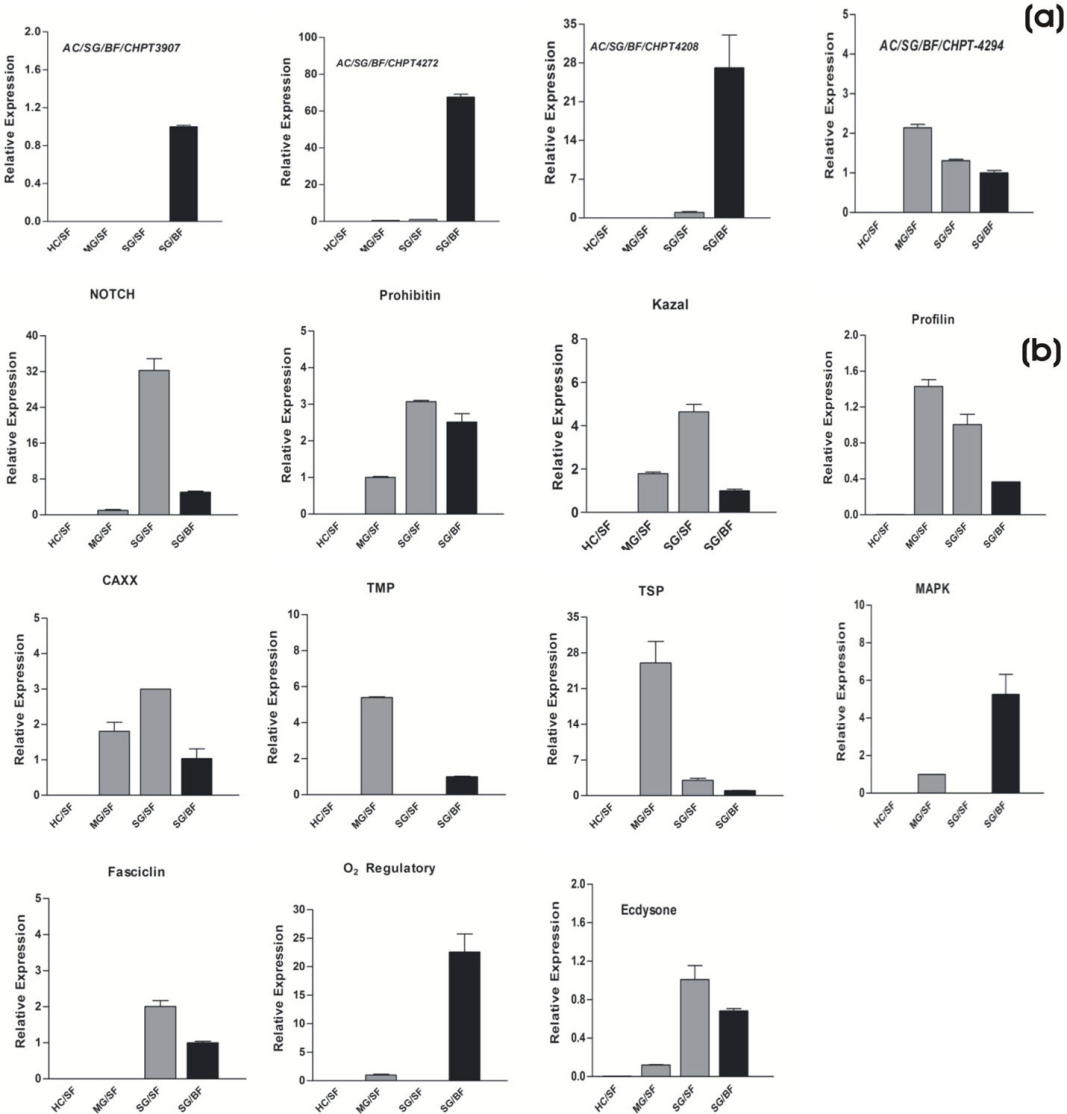
**Gene expression analysis of dual feeding associated other salivary proteins.** (A) Tissue-specific and differential regulation of three novel conserved hypothetical (CHPTs) salivary specific proteins possibly involved in sugar/blood feeding (transcripts are labeled with contigs number). (B) Tissue-specific relative expression analysis of the other salivary proteins of known functions (see Table 4), probably involved in cellular and physiological function of sugar/blood meal digestion or metabolism (HC, hemocyte; MG, midgut; SG, salivary gland; SF, sugar fed; BF, blood fed). Error bar represents standard deviation from three biological replicates.

To further predict possible function of other putative salivary transcripts involved in sugar/blood feeding, we identified and catalogued a pool of at least 15 salivary transcripts, which included (i) conserved hypothetical (CHPT) proteins, with unknown functions (ii) signaling pathway protein (iii) and other physiological functions (Table 4). Our real-time PCR analysis revealed that out of four selected CHPTs (all originated from blood fed salivary transcriptome), at least three transcripts (Fig. 6A) are exclusively induced in response to the blood meal in the salivary glands, suggesting their important role in blood feeding. Additionally, we also examined several other transcripts which expressed in the epithelial tissue of the digestive tract viz. midgut and salivary gland, probably associated with common physiological roles during food digestion (Fig. 6B).

**Table 4.**
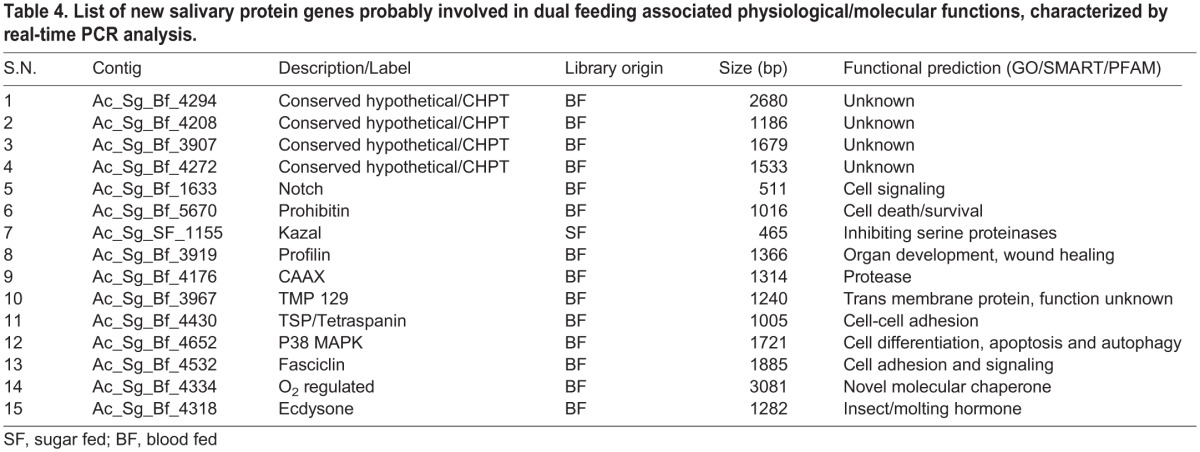
**List of new salivary protein genes probably involved in dual feeding associated physiological/molecular functions, characterized by real-time PCR analysis.**

### Salivary gene expression switching manages meal specific responses

**Fig. 7.**
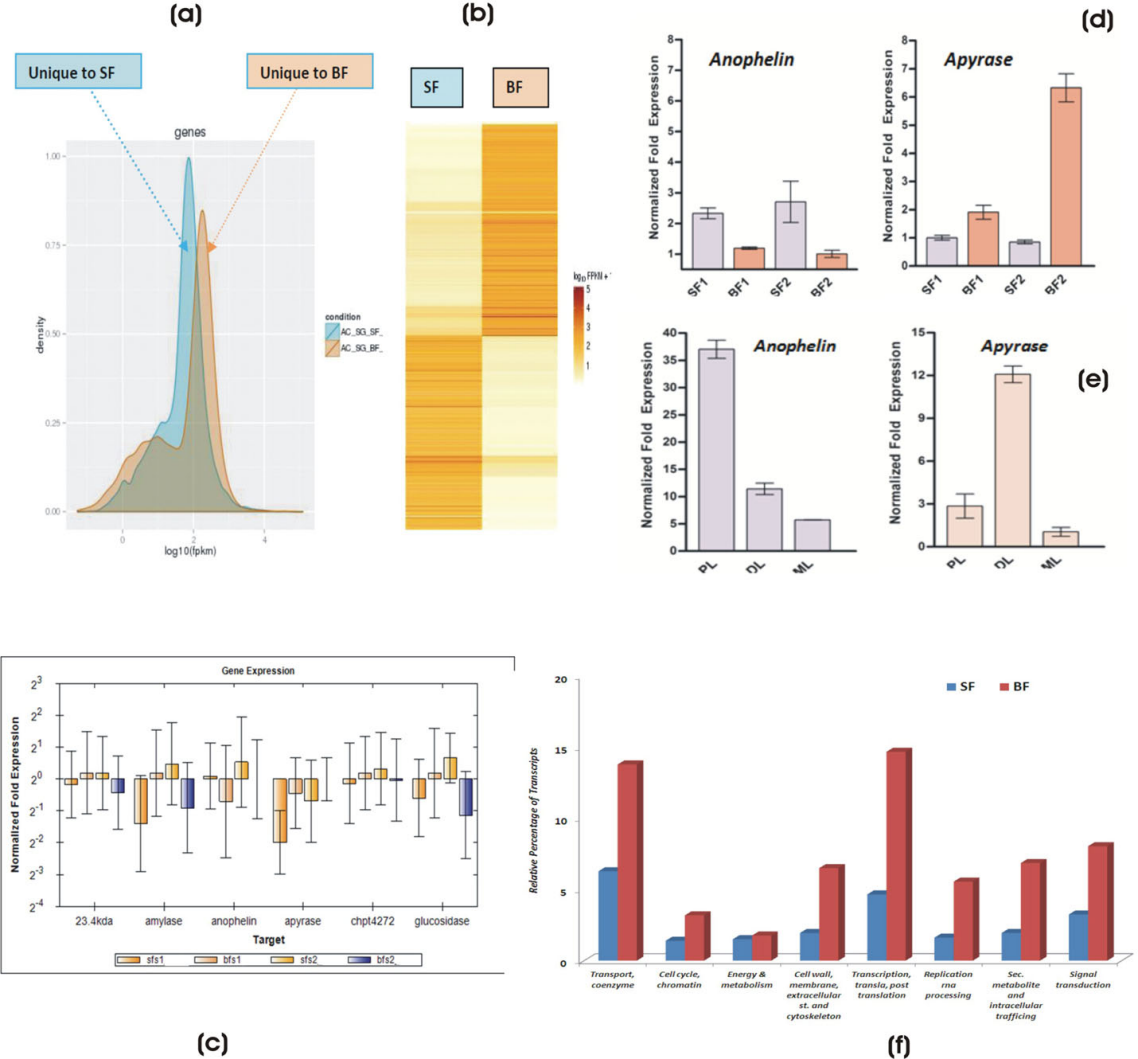
**Salivary gene expression switching in response** **to dual feeding.** (A) Read Density plot of the transcritome comparison showing unique contigs abundance restricted to feeding specific conditions of sugar feeding (blue) and blood feeding (orange). This analysis showed restricted expression of 1195 contigs to sugar feeding (12%) and 1021 contigs (10%) to blood feeding. See also supplementary material Table S5. Overlapping regions demonstrate commonly expressed genes. (B) Heat-map showing the global profiling comparison and distinct pattern of common salivary gene showing significant (*P*≤0.05) differential regulation. Relative gene abundance is defined by log10 of the normalized read number followed by Z-score transformation to visualize the expression level. Yellow indicates lower expression and red indicates higher expression. At least 17% of the transcriptome shows significant differential regulation of gene expression (*P*≤0.05), resulting in the expression alteration of 1767 contigss (847 blood fed and 920 sugar fed). (C) Real-time PCR-based verification of salivary gene expression in response to meal switching from sugar-blood-sugar-blood. (D) Real-time PCR-based validation of *Anophelin* and *Apyrase* (*P*≤0.05) regulation in response to meal specific switching; (E) Lobes specific expression of salivary *Anophelin* and *Apyrase* genes in blood fed mosquitoes (PL, Proximal lobe; DL, Distal lobe; ML, Median lobe). (F) Feeding specific switching results in the alteration of the molecular architecture of salivary glands: GO-Term based classification of salivary contigs significantly altered in response to blood feeding. SF, Sugar Fed; BF, Blood Fed; SFS1/SF1, Sugar Fed Series1; BFS1/BF1, Blood Fed Series1; SFS2/SF2, Sugar Fed Series2; BFS2/BF2, Blood Fed Series2. Error bar represents standard deviation from three biological replicates.

Next, to know the possible mechanism of how salivary glands facilitate and manage meal specific responses, we first examined the global changes in the molecular repertoire of salivary glands, occurring in response to naïve sugar fed status to first blood meal. We used merged contigs assembly to build reference database and performed digital gene expression (DGE) analysis by comparing each transcriptome according to the number of mapped reads (FPKM). To quantify the differences, we normalized the expression level of sugar fed (SF) and blood fed (BF) salivary transcriptome reads, before calculating the read ratio of SF to BF/fold change of relative expression. The read density plot (Fig. 7A) and heat map (Fig. 7B) analysis of DGE data showed meal specific restricted expression of unique tags to sugar (12%) or blood feeding (10%) as well as significant alteration of 17% tags (*P*≤0.05) in response to differential feeding (supplementary material Table S5).

Taken together, we hypothesize that mosquito may carry unique ability to switch on/off salivary gene expression, to manage meal specific responses. To further test this hypothesis, we examined the meal specific differential regulation in response to dual meal follow up from sugar-to-blood-to-sugar, by real-time PCR assay (see supplementary material Fig. S6 for technical overview). As expected, this analysis clearly showed a distinct pattern of expression switching, especially for *Anophelin* and *Apyrase* (*P*≤0.05), which predominantly express in distinct lobes of the salivary glands (Fig. 7C-E). GO term classification analysis also revealed significant up-regulation of salivary transcripts associated with transport, cytoskeleton, signaling and protein synthesis machinery (Fig. 7F), indicating that food specific switching may significantly alter molecular architecture of the salivary glands, supporting previous findings (Das et al., 2010). Interestingly, expression of energy/metabolism related genes did not alter during blood meal uptake, probably as mosquitoes do not fly and feed much immediately after blood meal (Goncalves et al., 2009).

Blood feeding is thought to have evolved independently several times among different arthropods (Lehane, 2005), imposing direct recruitment of the salivary products for a faster way to disrupt multiple homeostasis and inflammatory responses of the vertebrate hosts (Figueiredo et al., 2012; James, 2003). Thus, our data also provide an explanation to the recent observation of salivary protein depletion in response to blood meal (Sor-suwan et al., 2014), by the fact that adaptation of adult female mosquitoes to the vertebrate host might have favored the evolution of unique ability of salivary gene expression switching, to meet the dual feeding associated conflicting demands.

### Salivary glands undergo morphological and cellular changes in response to blood feeding

**Fig. 8.**
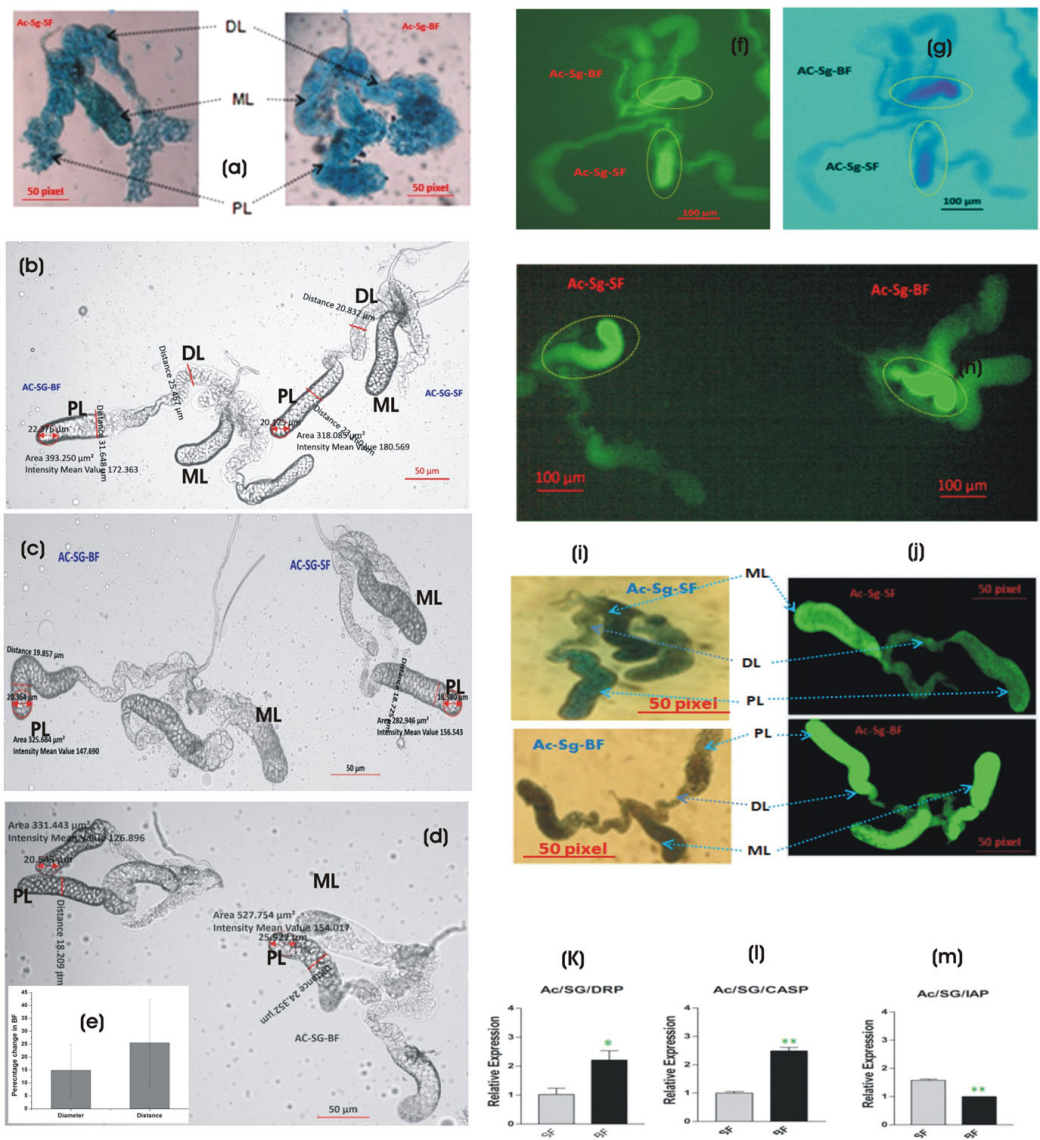
**Impact of blood meal on adult female mosquito salivary glands.** (A) Blood meal alters the morphological architecture, resulting in the swelling and extension of the salivary lobes. Nile blue stained glands observed under simple compound microscope (10× zoom) and captured with 7× mega pixels camera (Sony). (B-D) Phase contrast microscopy and (E) quantitative estimation of morphological features altered in response to first blood meal in the salivary glands (see text for detail). (F-J) TUNEL assay demonstrating apoptotic response in (F-H) the medial lobe (ML) (yellow circle) of the salivary glands and (I-J) in the distal and proximal lateral lobes (DL, PL) post blood meal. Loss of green color after staining with methylene blue is due to defragmentation of nuclear DNA. Note: image G is negative to image F with custom color background provided within the software for more better intensity resolution. (K-M) Relative expression of positive (DRP, draper; CASP, caspase) and negative (IAP, inhibitor of apoptosis) apoptotic marker genes in the salivary glands. Ac, Anopheles culicifacies, SG, salivary gland. (*P≤0.05; **P≤0.005). Error bar represents standard deviation from three biological replicates.

Our above data confirmed that the first blood meal uptake significantly alters the salivary molecular architecture; however, whether this first blood meal alters any morphological or cellular architecture remains unexplored. Therefore to trace any possible link with cellular events associated with meal specific switching, first we compared Nile blue stained sugar fed and immediate blood fed salivary gland, under simple microscope. Interestingly, the comparison showed that first blood meal causes irreversible alteration in the morphological architecture. It includes the extension, widening and swelling of the proximal, distal lateral and median lobes (Fig. 8A, supplementary material Fig. S8). Subsequent comparison between 3 days post first and second blood meal did not show further morphological alteration, suggesting that first blood meal results in major and permanent changes in the lobes architecture. However, whether, these changes have any advantages on subsequent blood meal and/or sugar meal acquisition, remain to be understood. A comparison of salivary glands of sugar fed and blood fed mosquitoes revealed that first blood meal exposure alters a minimum of 12–22% morphological features, measured in respect to the diameter and distances in proximal and distal lateral lobes of blood fed salivary glands (Fig. 8B-E, supplementary material Fig. S8).

Next, to track any cellular events associated with first blood meal, we revisited our comparative data and observed a significant change in the number of transcripts associated with autophagy (APHAGs) in response to blood feeding, (see Fig. 2B). We hypothesize that salivary glands may undergo an apoptotic event possibly to minimize the damage associated with blood meal uptake e.g. high pressure of blood flow, temperature switching 37°C to 28°C, multiple blood factors etc. Therefore, to test this hypothesis, first we performed TUNEL assay for sugar fed and immediate blood fed salivary glands. Within 5 min of incubation with labeling mixture, relatively higher fluorescence intensity could be observed in medial lobe of the blood fed salivary glands (Fig. 8F-H). Slightly, longer incubation for 30 min resulted in relative increase of brownish coloration (immuno staining; Fig. 8I) or green fluorescence (fluorescence labeling; Fig. 8J) in proximal and distal lateral lobes of the blood fed mosquito salivary glands. Taken together, data analysis of at least five independent experiments demonstrated that blood meal ingestion may cause an apoptotic event in each lobe of the mosquito salivary glands, probably to overcome the physiological challenges occurring in response to blood meal uptake.

The blood meal induced down regulation of apoptosis inhibitor (*AcIAP*; *P*≤0.005) and up regulation of *AcCaspase* (*P*≤0.005) as well as *AcDrpr* (*P*≤0.05) (an important phagocytic engulfment receptor homolog of *Drosophila Draper* (McPhee et al., 2010), further supported that blood meal induced apoptotic events are tightly controlled, for the successful removal of dying cells undergoing apoptosis in the salivary glands (Fig. 8K-M). However, whether the blood meal induced apoptosis also results in the regeneration and/or renewal of specific cells in the salivary glands remains to be clarified.

### Conclusion

Evolution and adaptation to dual feeding (sugar versus blood) behavior of adult female mosquito still remains a central question, therefore this knowledge can be critical to design vector borne disease management strategies. Comprehensive transcriptomic analysis under dual feeding status, provide initial evidence that mosquito salivary glands are able to encode meal specific diverse molecules. Comparative morphological, cellular and molecular analysis of blood meal impact supports the conclusion that adult female salivary glands are evolved with unique ability to manage meal specific responses.

## MATERIALS AND METHODS

Technical overview and work flow of the project is presented in supplementary material Fig. S1 document.

### Mosquito rearing

Cyclic colony of the mosquito *A. culicifacies* sibling species A, were reared and maintained at 28±2°C, RH=80% in the insectary fitted with a simulated dawn and dusk machine, essentially required for proper mating and feeding at NIMR (Adak et al., 1999). All protocols for rearing, maintenance of the mosquito culture were approved by ethical committee of the institute.

### cDNA library sequencing and assembly

Total RNA isolation and double stranded cDNA library preparation was done by PCR-based protocol as described previously (Dixit et al., 2009). For deep sequencing, Single-End RNA-seq libraries were generated for each sugar fed and blood fed salivary gland tissues by commercial service providers (NxGenBio Life Sciences, New Delhi, India). The tagged Single-End RNA-Seq libraries were diluted and pooled in equimolar concentrations and sequenced using TruSeq™ SBS Kit V2 on Illumina GAIIx (Illumina, San Diego, CA USA) for generating 1×36 bp single end sequencing reads. Following sequencing, the low quality bases were filtered or trimmed using in-house Perl scripts. All the bases, above Q20 phred score were used for further downstream analysis. *De-novo* transcriptome assembly was performed using Trinity assembler (Grabherr et al., 2011) with the default settings *k*-mer size of 25, minimum contig length of 150, unpaired reads option, average fragment length of 350 16 CPUs, with butterfly Heap space of 100G (allocated memory).

### Sequence annotation and digital gene expression (DGE) analysis

Following *de novo* clustering, CAP3 assembly using desktop cDNA annotation system was used (Guo et al., 2009), to build final contig dataset for functional annotation. The assembled contigs were subjected to similarity search against NCBI's NR database using the BLASTX algorithm (Altschul et al., 1990), with a cut-off E-value of ≤10^−3^ using BLOSUM62 matrix as well as GO annotation/Interproscan analysis using BLAST2GO (Conesa et al., 2005). Measurement of gene expression was computed using Trinity assembled denovo contigs of both sugar fed and blood fed salivary transcriptome, which were merged at 90% identity using CD-HIT algorithm for reference transcriptome buildup. The raw reads of sugar fed and blood fed transcriptomes were mapped on the reference using Tophat-Cufflinks pipeline (Trapnell et al., 2012). The mapped data was run through Cufflinks, Cuffcompare, Cuffdiff and CummeRbund pipeline to get the list of differentially expressed genes at *P value* of <0.05. Cuffdiff was used to generate differential expression results which were further used to plot Heatmap and differential expression plots by CummeRbund package. Relative expression of the genes was calculated by using Tophat-Cufflinks pipeline which generates value in FPKM (fragments per kilobase of exon per million fragments mapped). In the absence of replicates, we compared and estimated the tag dispersion, assuming two samples from different conditions with comparable gene expression levels. As both the samples contained RNA extracted from tissues of approximately 35 mosquitoes each and pooled to form one single sample, a quantifiable estimation of gene expression was expected with a minimum chance of aberrations. Our DESeq data analysis with single replicate was valuable to identify differentially expressed genes (Alaux et al., 2011; Wang et al., 2010), that were subsequently validated by large scale gene expression analysis by real-time PCR.

### PCR based gene expression analysis

The desired tissues viz. salivary glands, midgut and hemocyte (Rodrigues et al., 2010) or the whole body were directly collected in Trizol and processed for total RNA and first-strand cDNA synthesis as described previously (Dixit et al., 2011). For differential expression analysis, routine RT-PCR and agarose gel electrophoresis protocols were used. Relative gene expression was assessed by QuantiMix SYBR green dye (Biotool Biolabs, Madriad, Spain) in Biorad-iQ5 or CFX-96 Real-Time PCR machine. PCR cycle parameters involved an initial denaturation at 95°C for 15 min, 40 cycles of 10 s at 94°C, 20 s at 55°C, and 30 s at 72°C. Actin gene was used as an internal control in all qPCR measurements (supplementary material Fig. S7), where minimum two technical replicates were used in each real-time experiment. To better evaluate the relative expression, each experiment was performed in three independent biological replicates. The relative quantification results were normalized with internal control Actin gene and analyzed by 2^−ΔΔCt^ method (Livak and Schmittgen, 2001). Differential gene expression was statistically analyzed using Student *t*-test.

To demonstrate meal specific switching effect, out of 120 adult female (3–4 days old) mosquitoes, initially 20 pairs of salivary glands were collected from normal Sugar Fed_Series1 (SFS1); subsequently remaining mosquitoes were offered first blood meal and immediately pooled ∼20 pairs of SG from fully blood fed mosquitoes to complete Blood Fed_Series-1 (BFS1); Remaining fully engorged mosquitoes were kept back to normal sugar meal (Raisin) for next 72 h, for successful oviposition. From these remaining mosquitoes we collected second series of Sugar Fed (SFS2) and Blood Fed (BFS2) and monitored the relative expression by real-time PCR analysis (supplementary material Fig. S6). The primer sequences used in the study is listed in supplementary material Table S3.

### Salivary gland morphology and apoptotic assay

To detect the morphological alteration in response to blood feeding, salivary gland tissues collected from sugar fed or freshly blood fed were stained with Nile blue and observed under simple microscope. For quantitative estimations, both salivary gland tissues (sugar fed or blood fed) were dissected in phosphate buffer saline (PBS) and manually aligned in close approximation in such a way that images for both the salivary pair(s) could easily be observed and captured together for phase-contrast or fluorescent microscopy (ZeissScope.A1, Germany). If not successful in alignment, the images were captured individually and manually aligned in the image processing software (AxioCam ERc5s run with ZEN Lite Blue software) with multiple modules of image processing configurations and editing facilities. To maintain integrity and uniformity, all image manipulations and processing e.g. diameter, area, distances estimations and their labeling were manually done, within the given module environment of the software. Overall more than fourteen independent phase contrast microscopy experiments were performed, out of which only nine successful experiments provided recordable data and used for average percentage change estimation (percent change=average value in blood fed−average value in sugar fed×100/average value in sugar fed). Statistical analysis was carried out using Xcel Microsoft version 7 and OriginPro8.0 software.

To demonstrate salivary apoptosis event, TUNEL assay was performed either by labeling with fluorescein-dUTP or immuno staining, by using *In situ* Apoptosis Detection kit (Cat#MK500; Takara Bio-Inc.), as per manufactures instruction. Briefly, salivary glands were dissected from sugar and freshly blood fed 3–4 days old *A. culicifacies* mosquitoes and treated with kit provided buffers/components. After washing 2–3 times with sterile PBS for 10 min, salivary gland tissue was fixed with methanol containing 0.3% H_2_O_2_. Tissue was incubated with permeabilization buffer on ice for 2–5 min. Subsequently, tissues were incubated with pre-cooled labeling reaction mixture containing labeling safe buffer and TdT at 37°C for 5–30 min after PBS wash. Prepared mount was observed under fluorescent Zeiss scope A1 microscope attached with AxioCam ERc5s. For immuno staining immobilized tissue was washed with distilled water, stained with 3% methyl green and observed under light microscope.

### Accession numbers

The sequence data has been submitted to NCBI SRA database under following accession number: AC-SG-SF: SRR1952819 and AC-SG-BF: SRR1011070.

## Supplementary Material

Supplementary Material
