# Supplementary material for: Unraveling dual feeding associated molecular complexity of salivary glands in the mosquito Anopheles culicifacies
Source: Biol Open. 2015 Jul 10;4(8):1002–15. doi: 10.1242/bio.012294 (PMC4542284; doi:10.1242/bio.012294)
Supplement: Supplementary Material [file supp_4_8_1002__index.html]

Unraveling dual feeding associated molecular complexity of salivary glands in the mosquito Anopheles culicifacies — Unraveling dual feeding associated molecular complexity of salivary glands in the mosquito Anopheles culicifacies — Supplementary Material 

# Unraveling dual feeding associated molecular complexity of salivary glands in the mosquito *Anopheles culicifacies*

## BIO012294 Supplementary Material

- Supplementary Material
