# Supplementary material for: Unraveling dual feeding associated molecular complexity of salivary glands in the mosquito Anopheles culicifacies
Source: Biol Open. 2015 Jul 10;4(8):1002–15. doi: 10.1242/bio.012294 (PMC4542284; doi:10.1242/bio.012294)
Supplement: Supplementary Material [file supp_bio.012294_BIO012294supp.pdf]

Fig. S1. Technical overview of complete work flow for dual feeding associated mosquito Salivary Transcriptome Analysis

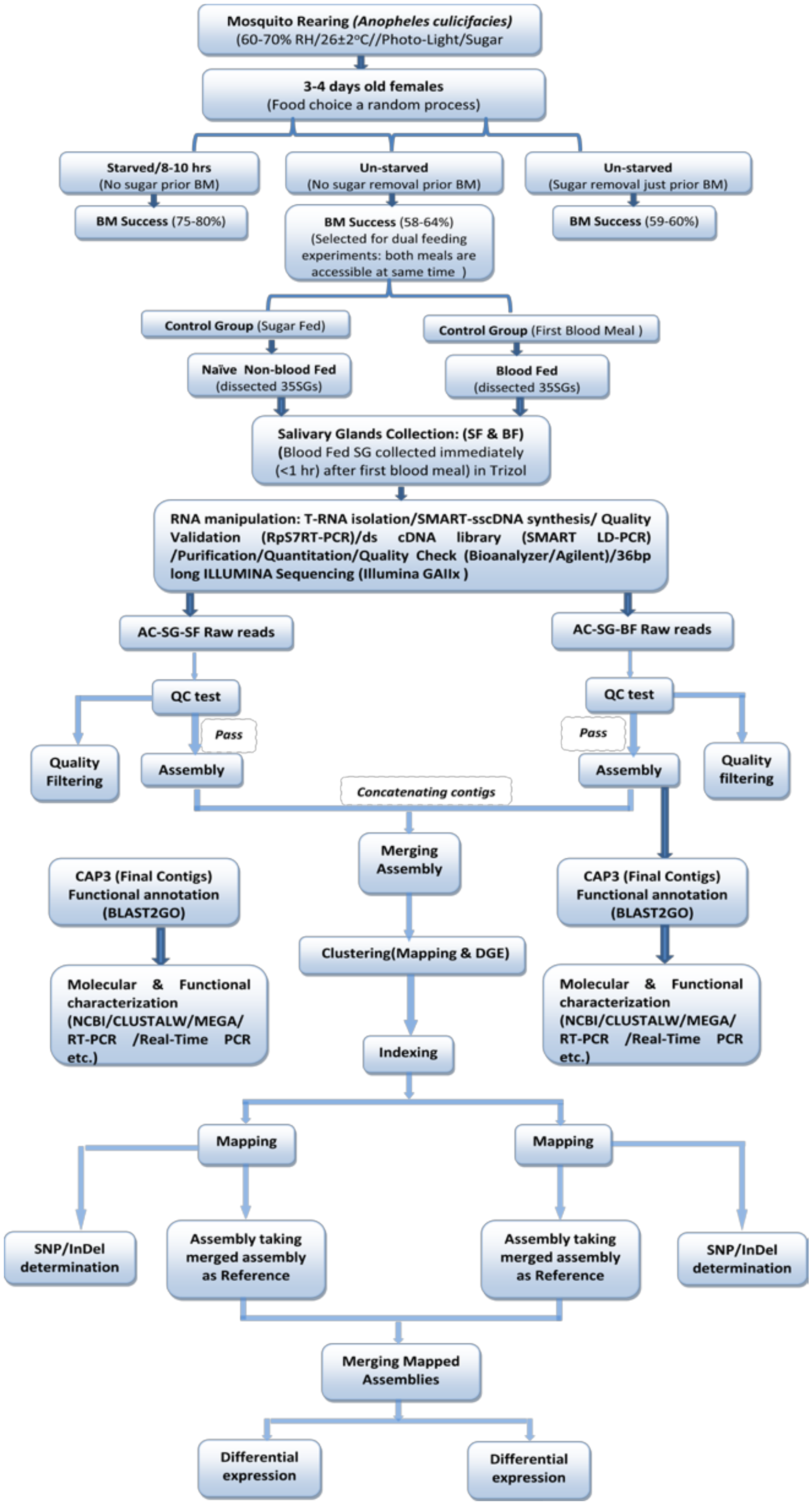

Fig. S2. Salivary Transcriptome Clustering & Assembly Stat. (i) Percentage of sequence size distribution; (ii) NR database similarity and percentage of data distribution according to size. SF, sugar fed; BF, blood fed.

(i)

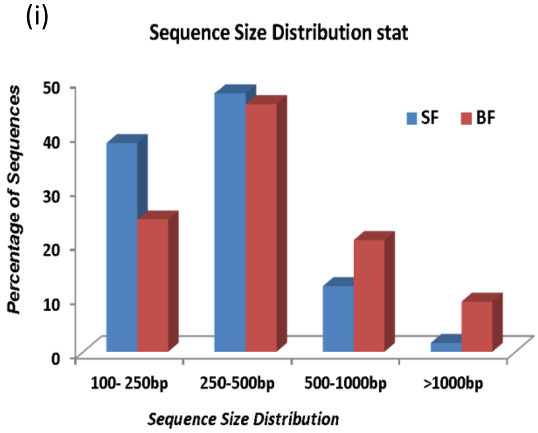

(ii)

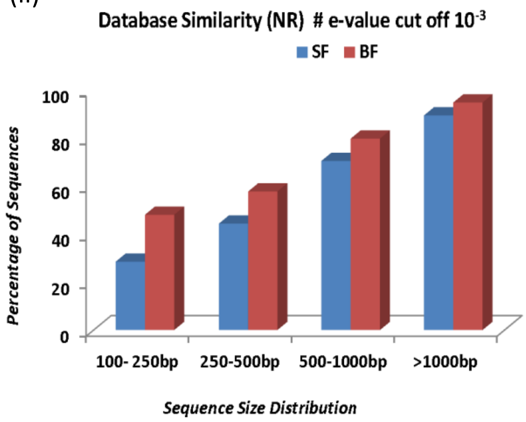

Fig. S3. Short reads based DENOVO assembly validation. Representative example of the 2182 bp long assembled cDNA encoding full length (576 amino acids) long secretary protein: (a) with all conserved domains of 5' nucleotidase super-family web-predicted; (b) Gene structure of AcAPY with other underlined motif features e.g. Amidation sites (Red) members; TKP (Violet) cAMP/cGMP dependent kinases (dark blue); N-Glycosylation (dark green); casein kinase (dark orange); protein kinase (pink) etc. (c) Phylogenetic relationship of AcAPY (Red arrow mark) with other characterized insect/mosquito Apyrases (Accession number has been presented with the name of the species), resulting clade formation with Anopheline and other blood feeding mosquito species (tree created at 1000 boot strap values); (d) Apyrase is a calcium-activated plasma membrane-bound enzyme that catalyses the hydrolysis of ATP to yield AMP and inorganic phosphate and abundantly expresses in the mosquito tissues as well during the development of the mosquito: RT-PCR based expression analysis of AcAPY (228bp): during development of the mosquito and different tissues of the mosquito *A. culcifacies.* Marker, 100bp Ladder; E, egg; L, larva; P, pupa; M, male; F, female; HC, hemocyte; MG, midgut; SG, salivary glands; SF, sugar fed; BF, blood fed. (e) RT-PCR validation of randomly selected un-annotated sequences from sugar fed library.

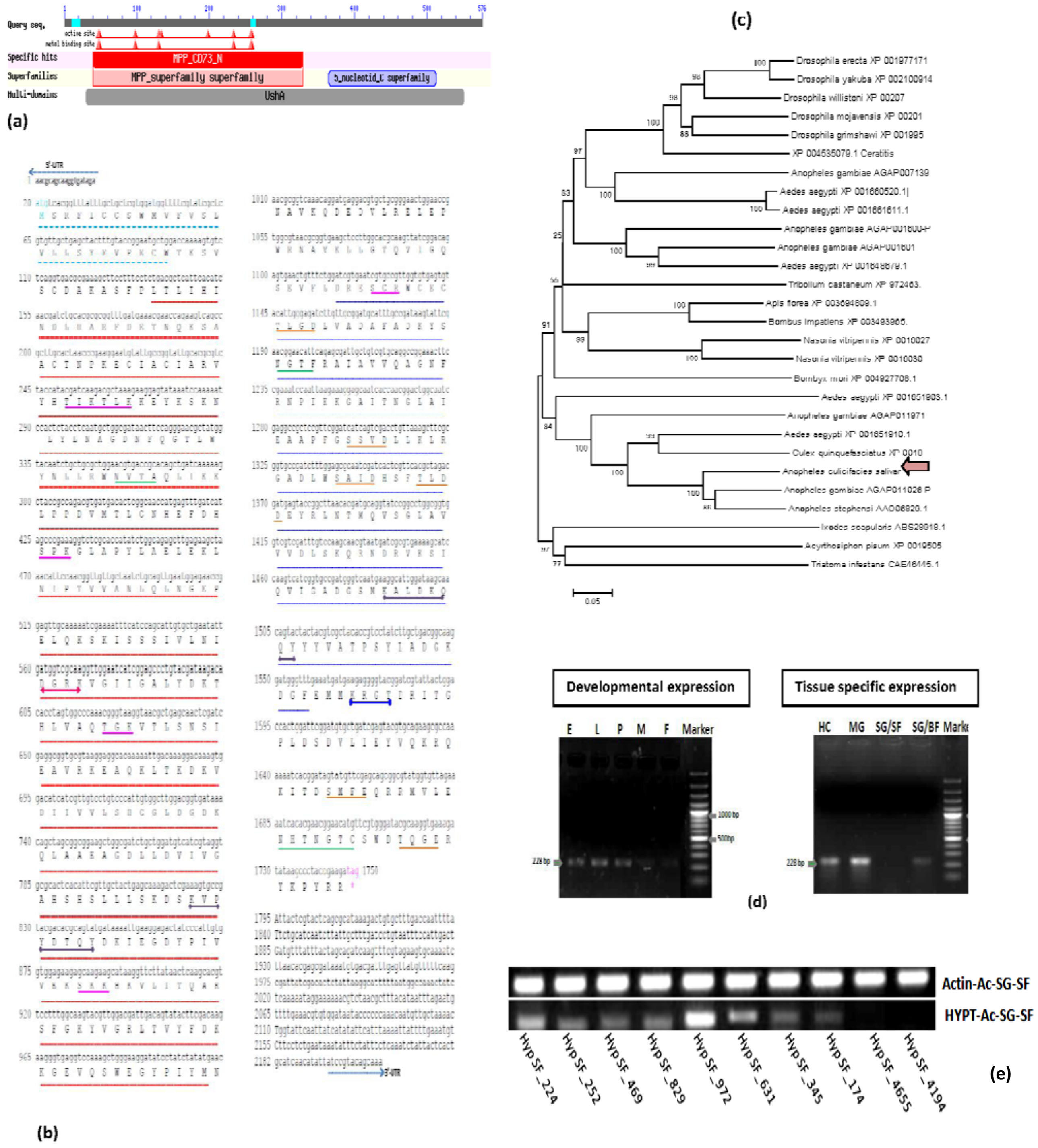

Query seq.
active site
metal binding site
Specific hits
MPP_CD73_N
Superfamilies
MPP_superfamily superfamily
5_nucleotid_C superfamily
Multi-domains
UshA
(a)
5'-UTR
3'-UTR
(b)
(c)
Drosophila erecta XP 001977171
Drosophila yakuba XP 002100914
Drosophila willistoni XP 00207
Drosophila mojavensis XP 00201
Drosophila grimshawi XP 001995
XP 004535079.1 Ceratitis
Anopheles gambiae AGAP007139
Aedes aegypti XP 001660520.1|
Aedes aegypti XP 001661611.1
Anopheles gambiae AGAP001601
Tribolium castaneum XP 972463.
Apis florea XP 003694809.1
Bombus impatiens XP 003493965.
Nasonia vitripennis XP 0010030
Bombyx mori XP 004927708.1
Aedes aegypti XP 001651903.1
Anopheles gambiae AGAP011971
Aedes aegypti XP 001651910.1
Culex quinquefasciatus XP 0010
Anopheles culicifacies saliva
Anopheles gambiae AGAP011026 P
Anopheles stephensi AAO06920.1
Ixodes scapularis ABS28919.1
Acyrthosiphon pisum XP 0019505
Triatoma infestans CAE46445.1
0.05
Developmental expression
E
L
P
M
F
Marker
1000 bp
500bp
228 bp
Tissue specific expression
HC
MG
SG/SF
SG/BF
228 bp
(d)
Actin-Ac-SG-SF
HYPT-Ac-SG-SF
HypSF_224
HypSF_252
HypSF_469
HypSF_829
HypSF_972
HypSF_631
HypSF_345
HypSF_174
HypSF_4655
HypSF_4194
(e)

Fig. S4. Mosquito salivary Transcriptome: Functional annotation and kinetics Stat (BLAST2GO analysis report). Distribution and comparison of mosquito salivary sequences by GO Annotation.

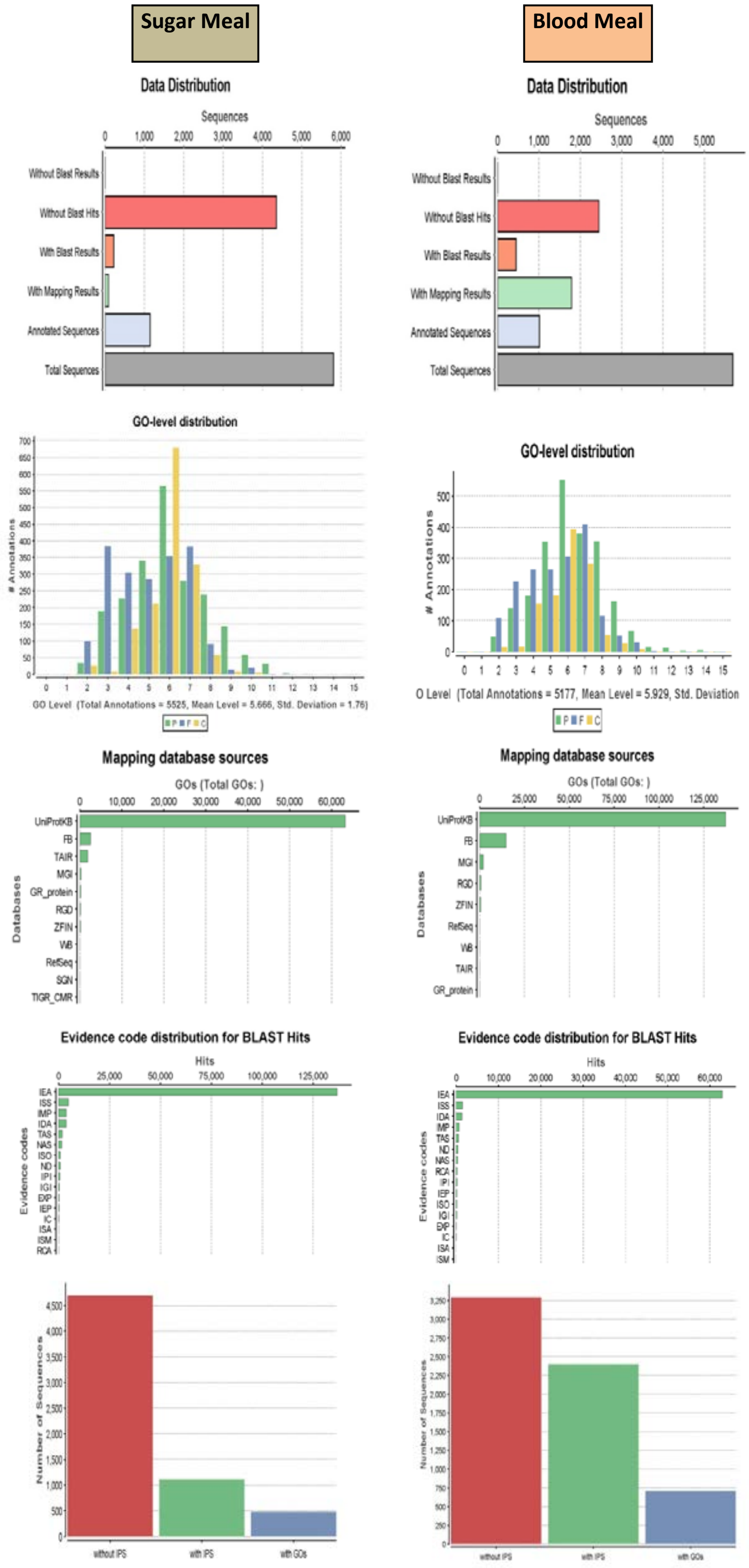

Fig. S5. BLASTX analysis of 23.4kDa salivary protein, showing restricted hits to the mosquito species in the NR database.

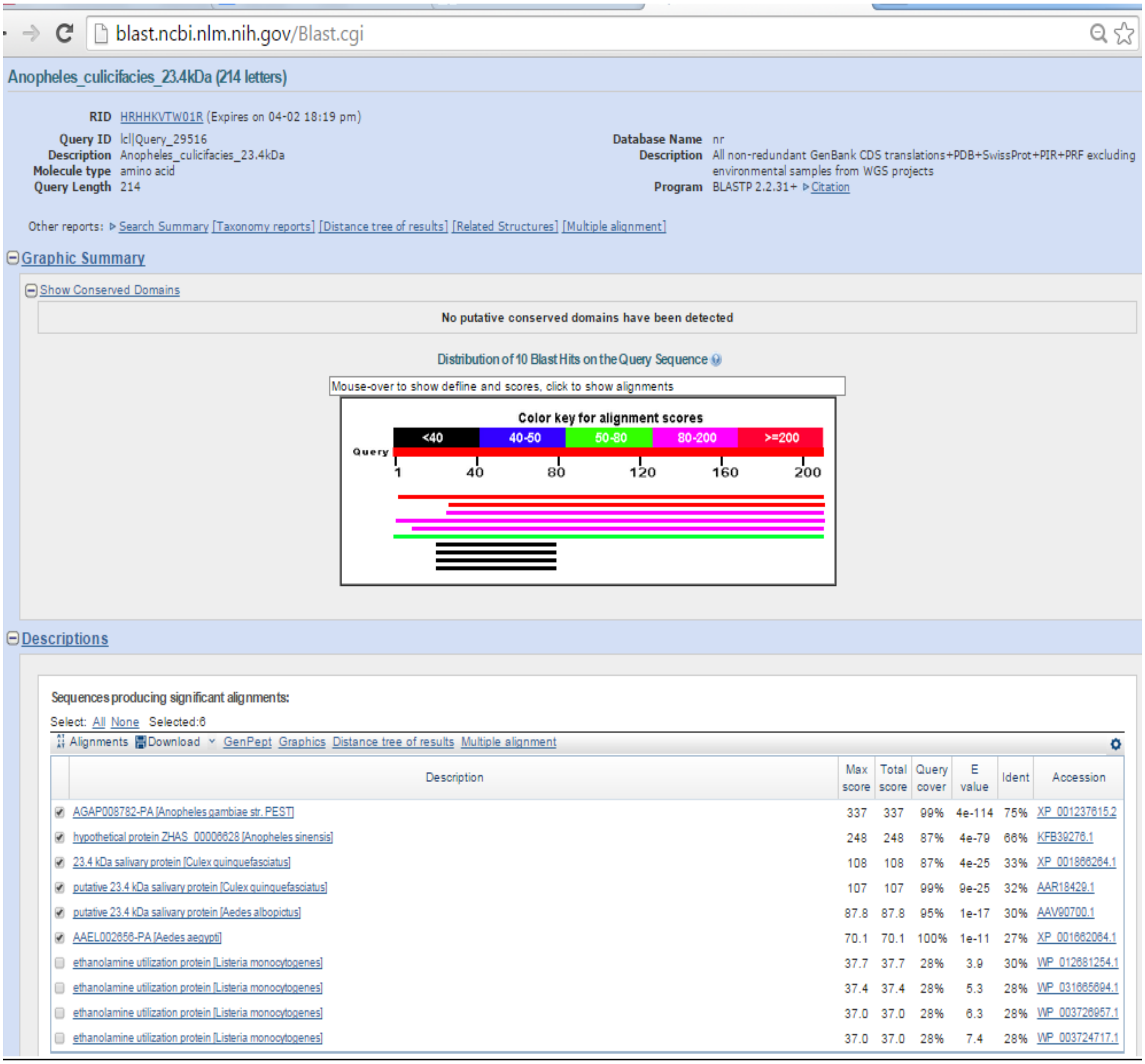

Fig. S6. Experimental design for sample collection to demonstrate dual feeding associated gene switching in the mosquito salivary glands.

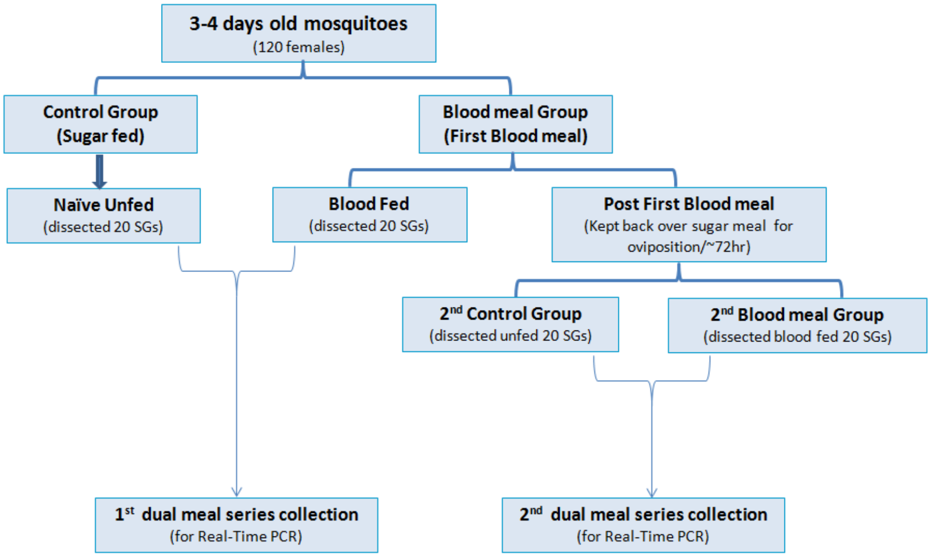

Fig. S7. To monitor the relative expression of salivary gene expression, we tested the suitability of actin vs. rpS7 gene as internal control for normalization. In our relative quantification analysis Actin gene showed lesser changes in the expression than rpS7, in the salivary glands of sugar fed vs. blood fed comparison. Thus we chose Actin as internal control in all relative gene expression analysis.

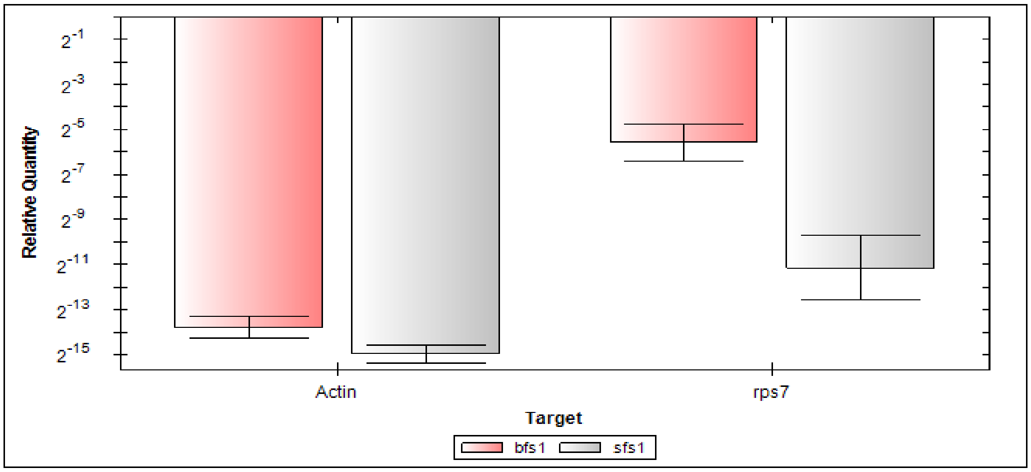

Fig. S8: Complete data of nine successful phase contrast microscopy experiments for quantitative estimation of morphological changes occurring in the salivary glands in response to blood meal. Bar chart represents the comparative change in the diameter, area, distance i.e. swelling/widening in the distal or proximal lateral lobes of the salivary glands.

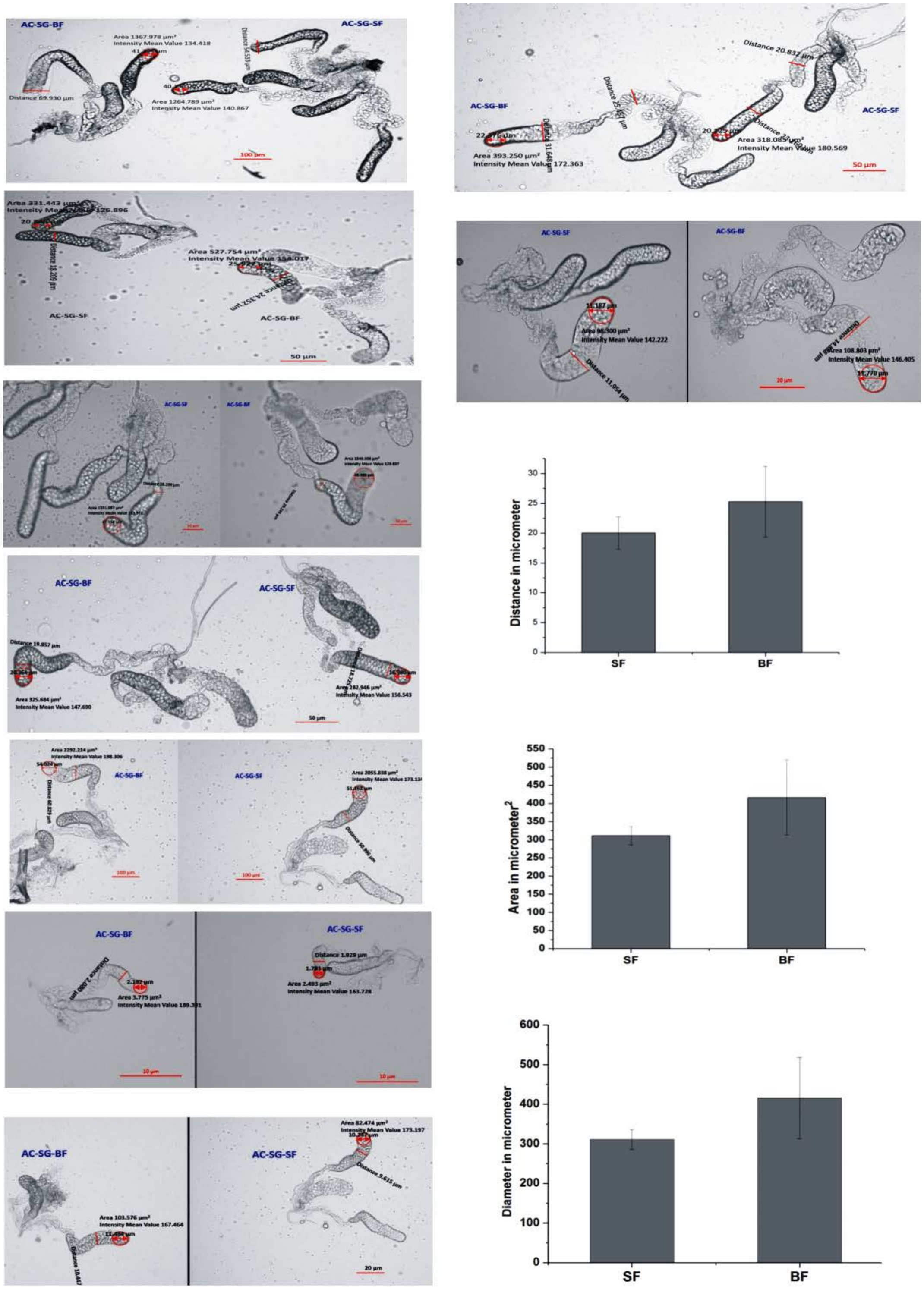

Table S1. Assembly validation: Initially to test and verify the quality of assembly, we selected and analyzed few longest contigs (1000-2500bp) encoding proteins, previously well characterized from the salivary transctriptomes of all most every mosquito sequenced so far. Table represents the molecular features of selected longer transcripts (>1000bp); encoding proteins of similar size as previously identified from almost every mosquito as reference sequence.

S.N.	Contig-ID	Label	Size (bp)	Amino acid no.	Nature	Function	RT-PCR Expression	
							Develop-mental<br>E L P M F	Salivary Gland<br>SF BF
1	AcSgBf-contig_3728	Salivary Apyrase	2182	582	Full Length	Inhibit platelet aggregation	+ + + + +	- +
2	AcSgBf-contig_3796	Alpha amylase	2274	731	Full Length	Hydrolyses alpha bonds of large, alpha-linked polysaccharides such as starch and glycogen	+ + + - +	+ +
3	AcSgBf-contig_3747	Glucosidase/ maltase precursor	1899	605	Partial	Break down of complex polysaccharides eg: starch and glycogen	+ + + + +	+ +
4	AcSgBf-contig_5372	Phospholipase	1909	512	Full Length	Inhibit the PAF induced platelet aggregation	+ + + + +	- +

Table S2. Differential regulation of salivary immune proteins in response to dual feeding.

S.N.	Label	AC-SG-BF_FPKM	AC-SG-SF_FPKM	log2 (fold change)	Regulation
1	DEF1 'Defensin'	37.2725	30923.1	9.69636	Down
2	Ag_IKK2 'IKKg'	1.68922	216.504	7.00189	Down
3	Ag_LYSC7 'LYSC'	0.813356	336.861	8.69405	Down
4	LysC-4 Ag_LYSC4 'LYSC'	0.829663	199.965	7.913	Down
5	Ag_CEC1 'Cecropin'	28.2624	5856.74	7.69507	Down
6	Ag_CEC2 'Cecropin'	45.5358	20751.7	8.83201	Down
7	Ag_CEC3 'Cecropin'	7.32717	7849.72	10.0652	Down
8	Ag_ML2 'ML'	2.28366	285.233	6.96465	Down
9	Ag_CLIPA14 'CLIPA'	1.18576	480.992	8.66406	Down
10	Ag_SCRASP3 'SCRA'	66.181	0.285879	-7.85487	Up
11	Ag_SCRAC1 'SCRA'	67.2465	0.266529	-7.97902	Up
12	Ag_SCRAC1 'SCRA'	119.045	0.243797	-8.93161	Up
13	Ag_REL2 'REL2'	217.091	1.68619	-7.00839	Up
14	Ag_REL2 'REL2'	68.852	0.083525	-9.68708	Up
15	Ag_HOP 'HOP'	90.2298	0.434407	-7.69841	Up
16	Ag_HOP 'HOP'	91.0458	0.165865	-9.10044	Up
17	Ag_REL1 'REL1'	79.6532	1.08974	-6.19168	Up
18	Ag_HPX4 'HPX'	402.03	3.2214	-6.96347	Up
19	Ag_HPX4 'HPX'	196.451	1.68349	-6.86658	Up
20	Ag_HPX4 'HPX'	48.268	0.104448	-8.85214	Up
21	Ag_TPX5 'TPX'	960.126	1.37981	-9.44261	Up
22	Ag_SCRB6 'SCRB'	49.2729	0.495833	-6.6348	Up
23	Ag_PPO3 'PPO'	42.7502	0.897587	-5.57373	Up
24	Ag_APG1 'APHAG'	91.271	0.154399	-9.20735	Up
25	Ag_APG7B 'APHAG'	65.8391	0.756251	-6.44394	Up
26	Ag_CASPAR 'CASPAR'	82.8807	0.305118	-8.08552	Up
27	Ag_CTLGA3 'CTLGA'	943.395	9.20755	-6.6789	Up

28	PGRPLD	318.647	4.08008	-6.28722	Up
29	Ag_GALE5 'GALE'	323.273	6.24282	-5.69441	Up
30	Ag_CLIPA15 'CLIPA'	47.0622	0.966843	-5.60514	Up
31	Ag_CLIPA6 'CLIPA'	53.8886	0.388076	-7.1175	Up
32	Ag_CLIPA7 'CLIPA'	197.358	0.688697	-8.16273	Up
33	Ag_CLIPA7 'CLIPA'	51.3964	1.26937	-5.33949	Up
34	Ag_CLIPA7 'CLIPA'	464.805	0.94036	-8.9492	Up
35	Ag_CLIPA7 'CLIPA'	288.927	2.71538	-6.73341	Up
36	Ag_CLIPA7 'CLIPA'	37.3	0.157826	-7.8847	Up
37	Ag_CLIPA7 'CLIPA'	173.227	0.384102	-8.81696	Up
38	Ag_CLIPA9 'CLIPA'	157.928	1.23003	-7.00442	Up
39	Ag_CLIPA9 'CLIPA'	76.2468	1.48048	-5.68654	Up
40	Ag_CLIPB1 'CLIPB'	2822.62	14.4489	-7.60993	Up

Table S3. List of primers and their sequences:

Sl. No.	Name -description	PRIMER SEQUENCE	
		**Forward**	**Reverse**
1.	FREP	5-'CCTGCTCGAAAACGTCCTAC-3'	5'-CGGTCAGCATACGGGTAGAT-3'
2.	PGRPLD	5'-TTCGAACCTTTCCTTTCACG-3'	5'-TTGAAAAACGCTTTCCGTCT-3'
3.	Salivary apyrase	5'-GGTCAATGAAGGCATTGGAT-3'	5'-AACATGTTCCGTTCGTGTGA-3'
4.	13.4kDa SGSP	5'-CCAAAATGCAACAGCATGTC-3'	5'-CATGCTTGCAGTAGCACCAT-3'
5.	SG1 LIKE	5'-AGCCCTTAGCAGGATGGTTT-3'	5'-AGCTTCTTCACGTCCTCCAA-3'
6.	23.4kDa SGSP	5'-GACTCTTACGACCGCAGAGG-3'	5'-GCTTGAACAGGAAGCTCTCG-3'
7.	PEROX	5'-CGCTCAAGAAGAGGAGATCG-3'	5'-TGTCGTGCACGGTACTAAGG-3'
8.	Alpha amylase	5'-CGTACAACGGTGTGTGGAAG-3'	5'-CTCAAGAAACACGCCTGACA-3'
9.	p38 MAPK	5'-CGCAACTTCAGTGACGTGTT-3'	5'-AGTGTCGAGGTCGGTTCATC-3'
10.	NOTCH	5'-TCGAAAGACGAATCGGAAAC-3'	5'-GCTTGTACGCGTCCCATTAT-3'
11.	O2 REG	5'-ATCTGACCAAGGATGCCAAC-3'	5'-CCCGATCTAGCATTCCCATA-3'
12.	LDL	5'-ACAAATTCACCGGCAAAGAC-3'	5'-CATTAGGCTGTCGCTCATCA-3'
13.	SAGLIN	5'-GAGCAGATGCAAACCAACCT-3'	5'-CTGGTTTGCCTTGGTTTTGT-3'
14.	PROFILIN	5'-CCACAGCAGCATCTCTAGCA-3'	5'-CCGTCTCGCTTCTTGTTTTT-3'
15.	CAAX	5'-TTTTGGGCTCCTGAGCTTTA-3'	5'-TGCATACGGGATTGAAACAA-3'
16.	CLIP B1	5'-AGAACGGTGCCGATTACAAC-3'	5'-AGCTGCTGTTTTCCGTCTGT-3'
17.	PROHIBITIN	5'-GAAAGCGGAACAGATGAAGC-3'	5'-GTTGGCCAGCTGGAAGATAA-3'
18.	TSP	5'-GCTTATAATCGGCCATCGAA-3'	5'-CTGCTGGTGATTGCACTGTT-3'
19.	CHPT	5'-CAAGGTGCTGCTAATCGTCA-3'	5'-CGCAAATGACACACCATTTC-3'
20.	OBP	5'-GACCGACTGTGCCAAGTGTA-3'	5'-GCCTGTTCGCGATACTTCTC-3'
21.	TMP129	5'-CGCCGATATTCGTCGTTAAT-3'	5'-GGATCTGGTACGGTGCTGAT-3'
22.	CHPT_4208	5'-CGCTCGGTTAGCAGAGTTTT-3'	5'-GACCCGATCAGGAAATGAGA-3'
23.	CHPT_3907	5'-TGATCTGGAGCGTCATCATC-3'	5'-GACGGTACTGTGTGGTGTCG-3'
24.	CHPT_4272	5'-CGATGCGTTGGTTTATTGTG-3'	5'-ATAGTGGTTCCGTGGTCTGC-3'
25.	Kunitz	5'-GGCACCCACCGTAGTAGAAC-3'	5'-GAATCGCATCGAGCTAAACC-3'
26.	kazal	5'-CGCCATTTCCGAATAGATGT-3'	5'-ACCAGACACGCCGGAGTTA-3'
27.	casps 5	5'-TCACAGGGCAGATCATCGTA-3'	5'-AATGTGGCGTTCGAAGAAGT-3'
28.	IAP6	5'-AAACCTGCCCCTTTTCATCT-3'	5'-CATCGGGCATGAAATCTTTT-3'
29.	Draper	5'-TCTTTACGATGCGTTGCTTG-3'	5'-AGTGGATACCATCCCCATCA-3'
30.	OBP_SF	5'-TTCCTACTCCGCGGTACAAC-3'	5'-ATGGATTGGCTTCACTGACA-3'
31.	Glucosidase	5'-CCGGGTTACGGTAGTTCAGA-3'	5'-CGTGGAACCGTACAAGGACT-3'
32.	Anophelin	5'-CTTCACCAAGATGCAGCAAA-3'	5'-TTTTCAAGGCTTTTGATACCG-3'
33.	Hypt_SF_4655	5’-GAGTATGAAGGACTGGTTGG-3’	5’-TTTCTCTCCCACTTCATCC-3’
34.	Hypt_SF_4194	5’- GGTGGTGGAGATTGCTATAA-3’	5’-ACGATCATATGGTCCAGAAC-3’
35.	Hypt_SF_174_Rev	5’- GATCCACAGGGTAACATCAC- 3’	5’-CTACAACGATCGCTCTTCA-3’
36.	Hypt_SF_224	5’TCAAGAACACGTACATCAGC 3’	5’GGAAATAGGGGAGTGAAATC 3
37.	Hypt_SF_252	5’ GAGATACCATCAACCGAATG 3’	5’ TAGCTGGAAAGCTCTTTGTC 3’
38.	Hypt_SF_345	5’ GATAGTGTGGGTGGTGGTTA 3’	5’ GTCACTGTCACCCGTTCTT 3’
39.	Hypt_SF_469	5’ TTTGCCAGAGAACTGAAGG 3’	5’CCACACTGTCGGACATTTAC 3’
40.	Hypt_SF_631	5’ GGGTACTGGGCATACAACA 3’	5’ CATGACTAACCCAACACCTC 3’
41	Hypt_SF_829	5’ CACTACTCCGGTACCATCCT 3’	5’ TCCGGTGTGTCTCTGTATGT 3’
42.	Hypt_SF_972	5’ AGATTGGTGGAAGATGTCG 3’	5’ GTAAGCCCGATAGACGTGTT 3’
43.	Actin	5'-TGCGTGACATCAAGGAGAAG -3'	5'-GATTCCATACCCAGGAACGA -3'
44.	RpS7	5'-TTGTTGAACTCGACCTCACG -3'	5'- ATCGCTATGGTGTTCGGTTC-3'

Table S4

Click here to Download Table S4

Table S5

Click here to Download Table S5
